# Feeding-induced resistance to acute lethal sepsis is dependent on hepatic BMAL1 and FXR signalling

**DOI:** 10.1038/s41467-021-22961-z

**Published:** 2021-05-12

**Authors:** Sarah S. Geiger, Javier Traba, Nathan Richoz, Taylor K. Farley, Stephen R. Brooks, Franziska Petermann, Lingdi Wang, Frank J. Gonzalez, Michael N. Sack, Richard M. Siegel

**Affiliations:** 1 Immunoregulation Section, Autoimmunity Branch, National Institute of Arthritis and Musculoskeletal and Skin Diseases (NIAMS), National Institutes of Health, Bethesda, MD USA; 2 Department of Biochemistry and Immunology, Trinity Biomedical Science Institute (TBSI), Trinity College Dublin, Dublin, Ireland; 3 Laboratory of Mitochondrial Biology and Metabolism, National Heart Lung and Blood Institute (NHLBI), National Institutes of Health, Bethesda, MD USA; 4 Departamento de Biología Molecular, Centro de Biología Molecular Severo Ochoa, Consejo Superior de Investigaciones Científicas-Universidad Autónoma de Madrid (CSIC-UAM), Madrid, Spain; 5 Biodata Mining and Discovery Section, Office of Science and Technology, NIAMS, NIH, Bethesda, MD USA; 6 Lymphocyte Cell Biology Section, Molecular Immunology and Inflammation Branch, National Institute of Arthritis and Musculoskeletal and Skin Diseases (NIAMS), National Institutes of Health, Bethesda, MD USA; 7 Laboratory of Metabolism, National Cancer Institute (NCI), National Institutes of Health, Bethesda, MD USA

**Keywords:** Sepsis, Bacterial infection

## Abstract

In mice, time of day strongly influences lethality in response to LPS, with survival greatest at the beginning compared to the end of the light cycle. Here we show that feeding, rather than light, controls time-of-day dependent LPS sensitivity. Mortality following LPS administration is independent of cytokine production and the clock regulator BMAL1 expressed in myeloid cells. In contrast, deletion of BMAL1 in hepatocytes globally disrupts the transcriptional response to the feeding cycle in the liver and results in constitutively high LPS sensitivity. Using RNAseq and functional validation studies we identify hepatic farnesoid X receptor (FXR) signalling as a BMAL1 and feeding-dependent regulator of LPS susceptibility. These results show that hepatocyte-intrinsic BMAL1 and FXR signalling integrate nutritional cues to regulate survival in response to innate immune stimuli. Understanding hepatic molecular programmes operational in response to these cues could identify novel pathways for targeting to enhance endotoxemia resistance.

## Abstract (web summary)

Time of day influences immune responses and lethality in response to LPS, with survival greatest at the beginning compared to the end of the light cycle. Here the authors show that feeding, rather than light, controls time-of-day dependent LPS sensitivity through the liver clock and hepatic FXR signalling.

## Introduction

Circadian rhythmicity in the innate immune response was first reported in 1960^1^ in the high-dose LPS model of sepsis, with mice exposed to LPS at zeitgeber time 0 (ZT0, beginning of the light cycle) having a survival advantage over those exposed at ZT12 (end of the light cycle). Since then, daily changes in lethality have been described upon stimulation with a variety of infammogenic materials^2,3^, and recent years have seen advances in understanding the molecular and cellular basis of this phenomenon, particularly building on the discovery of the cell-intrinsic circadian clock^4–7^. The myeloid clock has thereby received much attention and was proposed to be decisive in regulating mortality due to circadian variability in the production of inflammatory cytokines regulated by components of the cell-intrinsic clock, such as BMAL1^4,5^.

The last decade has also seen an increased understanding of metabolic cues in inflammation. While reversal of the feeding cycle can reprogramme circadian clock-controlled genes in the periphery^8,9^, the influence of the feeding compared to the light cycle on daily immune responses is not known. However, it should be noted that the circadian machinery integrates multiple environmental factors, such as the feeding and sleep–wake cycles that align under natural conditions, making it impossible to decipher the contributions of a single zeitgeber (an environmental signal that can regulate the internal clock) without specifically adjusted experimental settings. It is therefore likely that other factors will influence the host response to acute inflammatory stimuli beyond the light cycle. Indeed, metabolic cues such as prolonged fasting and the Farnesoid X-receptor, a component of the feeding-regulated bile acid cycle have been recently shown to influence inflammatory responses and survival in sepsis models^10,11^.

Importantly, sepsis is a leading cause of death in intensive care units and patient care is currently solely supportive due to a lack of effective therapies. Although inflammation is the foundation of sepsis, the exact cause of mortality is not clear^12^ and previous efforts using anti-cytokine antibodies, such as anti-TNF, anti-IL-1 and anti-IL-6, have remained unsuccessful in humans^13–15^. Understanding novel aspects and pathways is therefore urgently needed to find new targets to enhance sepsis survival. In this study, we reveal a critical role for the feeding cycle in regulating survival in the LPS model of sepsis. This feeding-cycle regulated susceptibility depends on BMAL-1 and FXR signalling in hepatocytes, identifying potential new targets for therapeutic intervention.

## Results

### Lethality in the LPS model of sepsis follows the feeding, not the light cycle

**Fig. 1:**
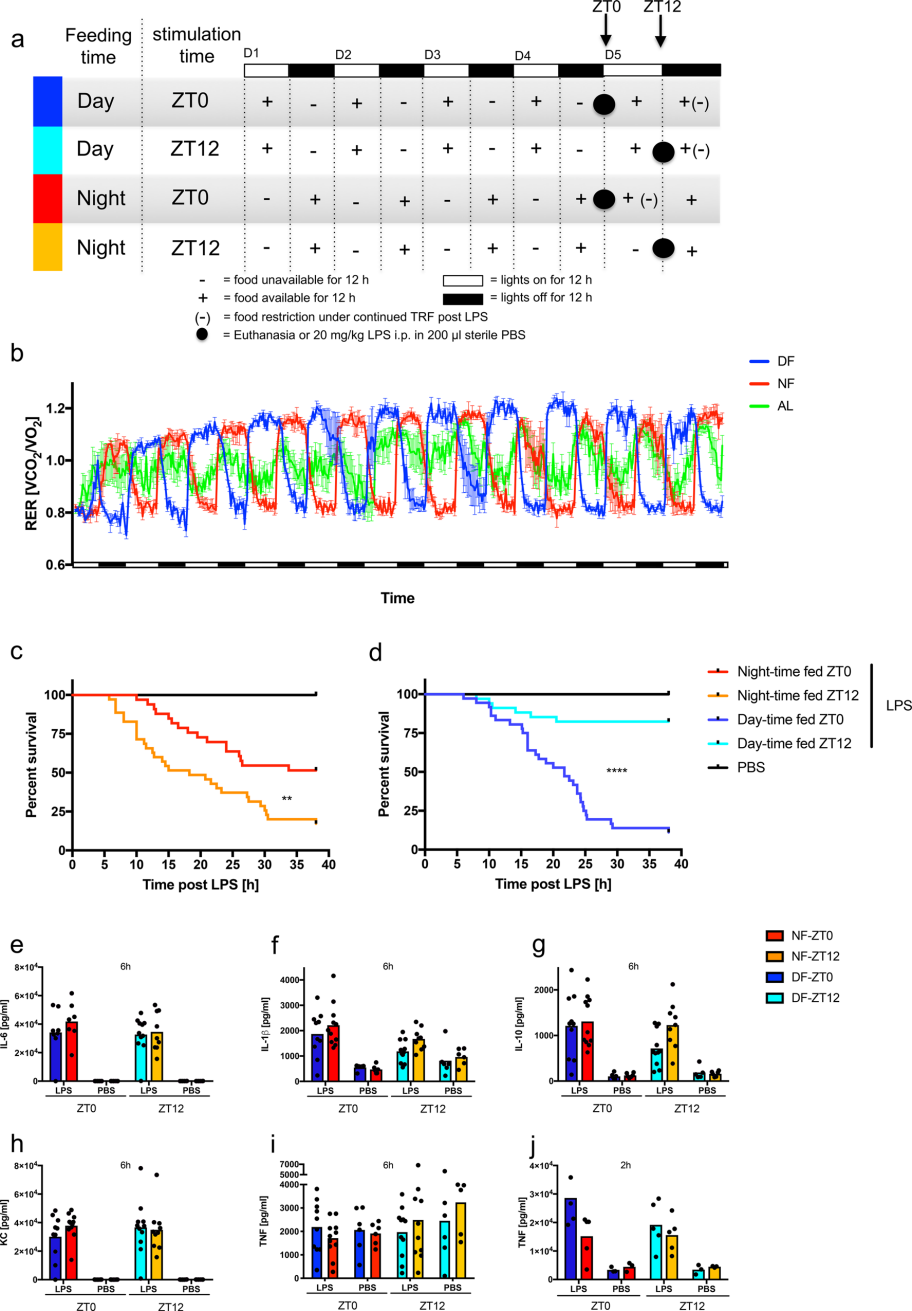
Feeding, rather than light controls susceptibility to LPS. **a** Graphic of the experimental setup illustrating light and feeding cycles. **b** Respiratory exchange ratio (RER) in female TRF mice over time housed in metabolic cages. Black bars indicate dark phases. DF: Food available during light phase only, NF: Food available during dark phase only, AL: Ad Lib, food available continuously. Representative of 7 individual experiments. *N* = 4. Data are presented as mean values ± SEM. Full statistical analysis using CalR in Supplementary Table 1. RER values min: DF = 0.65 NF = 0.67 AL = 0.71, max: DF = 1.32 NF = 1.28 AL = 1.23. **c**, **d** Morbidity in the night-time (**c**) or day-time fed (**d**) C57BL/6J and *Bmal1*^fl/fl^ mice following 20 mg/kg LPS i.p. administered at ZT0 or ZT12 on day 5 of TRF with the indicated schedule. Statistical analysis according to Mantel–Cox: NF 0 vs. 12: *p* = 0.0015, DF 0 vs. 12: *p* < 0.0001, ZT0 NF vs. DF: *p* = 0.0004, ZT12 NF vs. DF: *p* < 0.0001, DF-ZT0 vs. NF-ZT12: *p* = 0.8376, DF-ZT12 vs. NF-ZT0: *p* = 0.0143. Experimental numbers: DF-ZT0-LPS *N*_female_ = 20 *N*_male_ = 16, NF-ZT0-LPS *N*_female_ = 21 *N*_male_ = 12, DF-ZT12-LPS *N*_female_ = 20 *N*_male_ = 14, NF-ZT12-LPS *N*_female_ = 21 *N*_male_ = 14, DF-ZT0-PBS *N*_female_ = 11 *N*_male_ = 4, NF-ZT0-PBS *N*_female_ = 11 *N*_male_ = 5, DF-ZT12-PBS *N*_female_ = 10 *N*_male_ = 5, NF-ZT12-PBS *N*_female_ = 8 *N*_male_ = 6. **e**–**j** Serum cytokines 6 h (**e**–**i**) or 2 h (**j**) after LPS stimulation (20 mg/kg) of female mice on day 5 of TRF at ZT0 or ZT12 as indicated. Box indicates mean. No significance between LPS-treated samples within feeding (ZT0 vs. ZT12) or stimulation time (DF vs. NF) according to two-way ANOVA and Sidak’s multiple comparisons. Experimental numbers: **e** DF-ZT0-LPS *N* = 8, NF-ZT0-LPS *N* = 7, DF-ZT12-LPS *N* = 11, NF-ZT12-LPS *N* = 9, *N*(PBS) = 6. **f**–**i** DF-ZT0-LPS N = 10, NF-ZT0-LPS *N* = 11, *N*(PBS) = 6. **h**, **i** DF-ZT12-LPS *N* = 11, NF-ZT12-LPS *N* = 10, *N*(PBS) = 6. **f** DF-ZT12-LPS *N* = 10, NF-ZT12-LPS *N* = 9, N(PBS) = 6. **g** DF-ZT12-LPS *N* = 11, NF-ZT12-LPS *N* = 9, DF-ZT12-PBS *N* = 6, NF-ZT12-PBS *N* = 5. **j** *N*(LPS) = 5, *N*(PBS) = 3.

To dissociate the role of the feeding from the light cycle in LPS susceptibility, we studied mice under time-restricted feeding (TRF) conditions. Food access was restricted to either the dark phase (night-time fed, NF), the predominant feeding time for nocturnal species, or the light phase (day-time fed, DF), where food and light cycles become dissociated (Fig. 1a). As expected, activity paralleled the light cycle (Supplementary Fig. 1a), and night-time feeding closely approximated feeding patterns of ad libitum food availability (Supplementary Fig. 1b). Food consumption and activity levels were comparable between groups (Supplementary Fig. 1a, b) and the respiratory exchange ratio (RER) was >1 in the fed state, reflecting a switch to glucose utilization, decreasing to ~0.8, indicative of fatty acid oxidation, with food-deprivation (Fig. 1b). The RER thus closely paralleled the feeding rather than the light cycle. TRF mice displayed similar maximal and minimal RER, while differences remained less pronounced in ad libitum-fed animals (Fig. 1b).

We then challenged mice conditioned for 4 days to NF or DF schedules with LPS at ZT0 vs. ZT12 (Fig. 1a). Mice were monitored frequently for behavioral changes due to sepsis and euthanized at pre-specified morbidity (Supplemental methods). Congruent with the previous reports^1^, lethality was greater in NF animals following the LPS challenge at ZT12 (Fig. 1c). Strikingly, in DF mice, susceptibility was reversed, with 87% mortality following ZT0 LPS administration versus 82% survival following ZT12 administration (Fig. 1d). While LPS susceptibility, in concordance with previous reports^16^, was generally less pronounced in male than female mice, feeding time-dependent sensitivity was evident in both sexes (Supplementary Fig. 1e–h). The majority of subsequent experiments were therefore performed on mixed populations (female data points in black and males in red), as has been done in comparable studies^17^. The regulation of susceptibility by feeding cycle persisted whether the food was provided ad libitum after LPS or remained restricted according to the prior feeding schedule (Supplementary Fig. 1c, d), indicating that the outcome was not determined by the availability of food post-stimulation. It should be noted, that in concordance with previous findings^18^, food intake was significantly blunted following LPS stimulation (Supplementary Fig. 1i). As food intake remained voluntary, this phenotype may not be applicable to studies investigating anti-anorexic forced feeding effects^19^. Systemic cytokine production, a measure of the inflammatory state, was not correlated with food-regulated LPS susceptibility, with similar levels of pro-inflammatory cytokines IL1β, IL6, TNF, the chemokine KC, and the counter-regulatory cytokine IL10, regardless of the feeding schedule at baseline and following LPS (Fig. 1e–j, Supplementary Fig. 1j–o). Soluble CD14, a biomarker for systemic LPS exposure, was also similar between feeding groups at baseline (Supplementary Fig. 1p) and after LPS challenge (Supplementary Fig. 1q). Thus, the feeding cycle influence on acute mortality after LPS appears not to be dependent on inflammatory cytokine response or LPS bioavailability.

### Profound hypoglycaemia is associated with increased LPS sensitivity

**Fig. 2:**
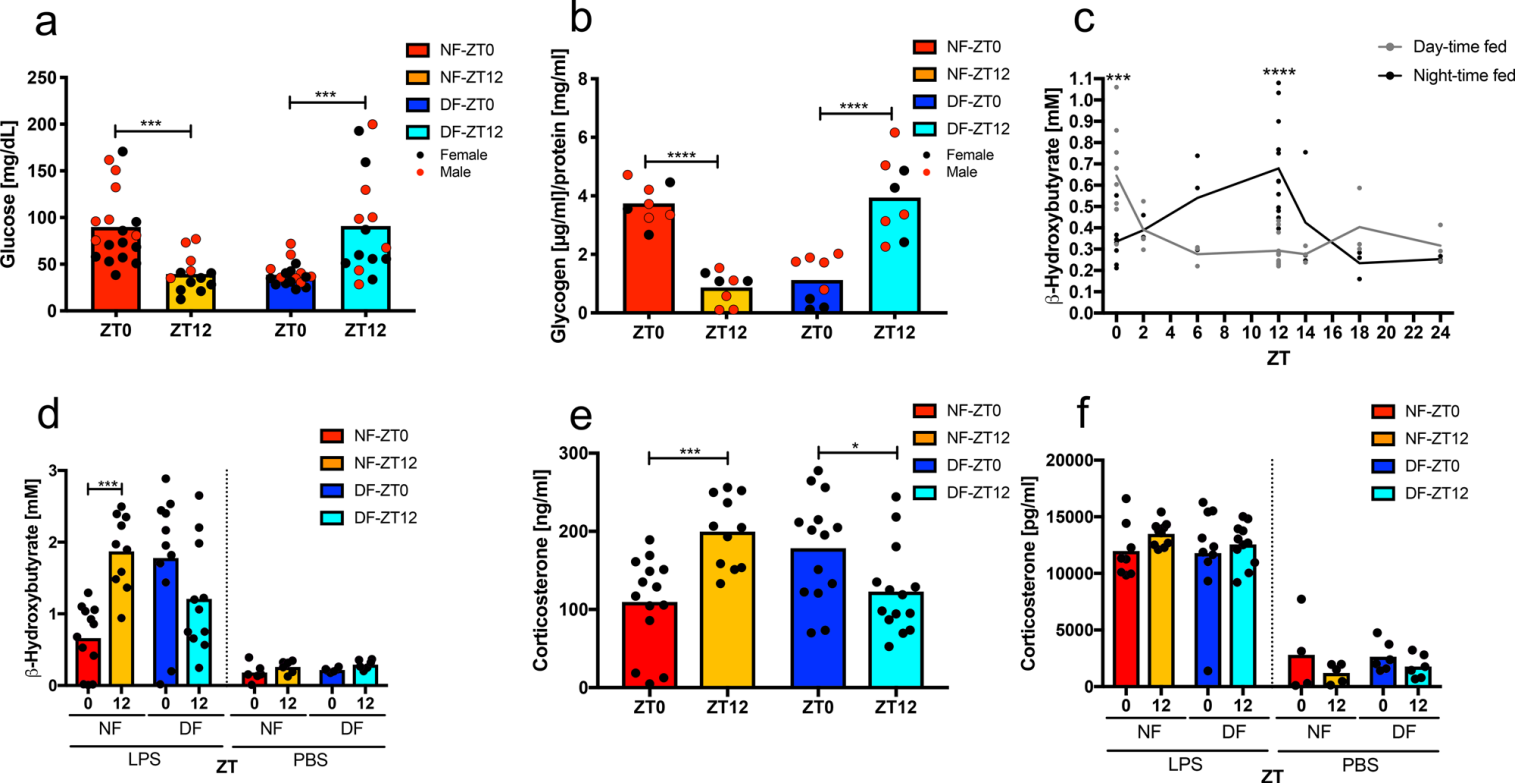
Glucose metabolism parallels mortality. **a** Serum glucose (*p* < 0.0001) 6 h post LPS (20 mg/kg) stimulation on day 5 of TRF. Female in black, male in red. NF-ZT0 *N*_female_ = 11 *N*_male_ = 7, NF-ZT12 *N*_female_ = 8 *N*_male_ = 5, DF-ZT0 *N*_female_ = 11 *N*_male_ = 7, DF-ZT12 *N*_female_ = 9 *N*_male_ = 7. Box indicates mean. Statistical significance according to two-way ANOVA and Sidak’s multiple comparison (ZT0 vs. ZT12) as indicated. *p*_NF_ = 0.0002, *p*_DF_ = 0.0007. Interaction *F*_(1,66)_ = 34.21, *p* < 0.0001, ZT *F*_(1,66)_ = 0.09264, ns, Feeding *F*_(1,66)_ = 0.03347, ns. **b** Liver glycogen content on day 5 of TRF measured at ZT0 or ZT12. Female in black, male in red. *N*_female_ = 3 *N*_male_ = 5. Box indicates mean. Statistical significance according to two-way ANOVA and Sidak’s multiple comparisons (ZT0 vs. ZT12) as indicated. Interaction *F*_(1,28)_ = 78.25, *p* < 0.0001, ZT *F*_(1,28)_ = 0.006643, ns, Feeding *F*_(1,28)_ = 0.4853, ns. ****p < 0.0001. **c** Baseline serum levels of β-hydroxybutyrate (BHB) at the indicated ZT on day 5 of day-time (grey) or night-time feeding (black) (Interaction *F*_(6,61)_ = 10.31, *p* < 0.0001, ZT *F*_(6,61)_ = 2.704, *p* = 0.0215, Feeding *F*_(1,61)_ = 0.717, *p* = 0.4004), *N*_ZT0_ = 10, *N*_ZT2_ = 3, *N*_ZT6_ = 3, *N*_DF-ZT12_ = 13, *N*_NF-ZT12_ = 12, *N*_ZT14_ = 3, *N*_ZT18_ = 3, *N*_ZT24_ = 3. Each dot represents an individual data point and the line represents the mean. Females only. Statistical analysis using two-way ANOVA and Sidak’s multiple comparisons (NF vs. DF). ***p = 0.0003 ****p < 0.0001. **d** BHB serum levels 6 h post LPS on day 5 of TRF at ZT0 or ZT12 in female mice. DF-ZT0-LPS *N* = 11, NF-ZT0-LPS *N* = 12, DF-ZT12-LPS *N* = 11, NF-ZT12-LPS *N* = 11, DF-ZT0-PBS *N* = 6, NF-ZT0-PBS *N* = 6, DF-ZT12-PBS *N* = 6, NF-ZT12-PBS *N* = 6. Mean is indicated by the box. Statistical analysis using two-way ANOVA (LPS and PBS separately) and Sidak’s multiple comparison (ZT0 vs. ZT12) as indicated. LPS: Interaction *F*(1,40) = 18.29, *p* < 0.0001, ZT *F*_(1,40)_ = 2.357, ns, Feeding *F*_(1,40_) = 1.184, ns. PBS: Interaction *F*_(1,20)_ = 0.001053, ns, ZT *F*(1,20) = 5.015, *p* = 0.0367, Feeding *F*_(1,22)_ = 0.9777, ns. ***p = 0.0004. **e**, **f** Serum corticosterone on day 5 of TRF at baseline (**e**) (Interaction *F*(1,49) = 20.36, *p* < 0.0001, ZT *F*_(1,49)_ = 1.159, ns, Feeding *F*_(1,49)_ =0.05739, ns, *p = 0.0305 ***p = 0.0007, DF-ZT0 *N* = 14, DF-ZT12 *N* = 14, NF-ZT0 *N* = 14, NF-ZT12 *N* = 11) or 6 h post LPS (20 mg/kg)/PBS control stimulation (**f**) (LPS and PBS ns, DF-ZT0-LPS *N* = 10, DF-ZT12-LPS *N* = 11, NF-ZT0-LPS *N* = 8, NF-ZT12-LPS *N* = 10, DF-ZT0-PBS *N* = 6, DF-ZT12-PBS *N* = 6, NF-ZT0-PBS *N* = 4, NF-ZT12-PBS *N* = 5) in female mice. Box indicates mean. Statistical analysis using two-way ANOVA (LPS and PBS separately) and Sidak’s multiple comparison (ZT0 vs. ZT12) as indicated.

LPS additionally induces metabolic reprogramming, including a shift to glycolysis^20^. As systemic hypoglycaemia can be lethal in the absence of compensatory gluconeogenesis^21^, we studied glucose metabolism in LPS-exposed mice after different feeding schedules. Mice susceptible to LPS (NF-ZT12, DF-ZT0) developed profound hypoglycaemia of <50 mg/dl, significantly less than mice in the resistant groups, which averaged 100 mg/dl (Fig. 2a). Kinetic analysis showed that blood glucose began falling as early as 2 h after LPS challenge, and in animals resistant to LPS, began to rise after 6 h (Supplementary Fig. 2a). TRF itself was not sufficient to affect baseline glucose levels (Supplementary Fig. 2b), but fasted groups (NF12 and DF0) had significantly depleted liver glycogen stores (Fig. 2b), which contribute to ineffective maintenance of glucose levels after LPS challenge. Fasting also induced ketogenesis, resulting in the production of β-hydroxybutyrate (BHB), which was reported to inhibit NLRP3 inflammasome activation^22^. In TRF mice, BHB increased during the food-restricted phase in DF and NF groups (Fig. 2c). LPS, independent of feeding status, further induced BHB, with higher levels at LPS-susceptible time points (Fig. 2d). This supports the notion of metabolic distress after LPS challenge in susceptible groups and shows that elevated BHB is not sufficient to reduce mortality or inflammatory cytokine levels in this model. Endogenous corticosteroids influence diurnal cycles of cytokine production^23^ and might also protect mice from LPS challenge. However, peak serum levels of corticosterone, the principal active circulating corticosteroid in mice, was found at ZT12 in nocturnally fed mice, and ZT0 in DF mice, when susceptibility to LPS is the greatest (Fig. 2e). As expected, glucocorticoids rose after the LPS challenge but were not different between feeding groups and time of stimulation nor the post-stimulation feeding regimen (Fig. 2f), showing that endogenous corticosteroids are not likely a key protective factor in food-cycle controlled LPS-induced mortality.

### The liver-intrinsic circadian clock rapidly adapts to changes in the feeding cycle

**Fig. 3:**
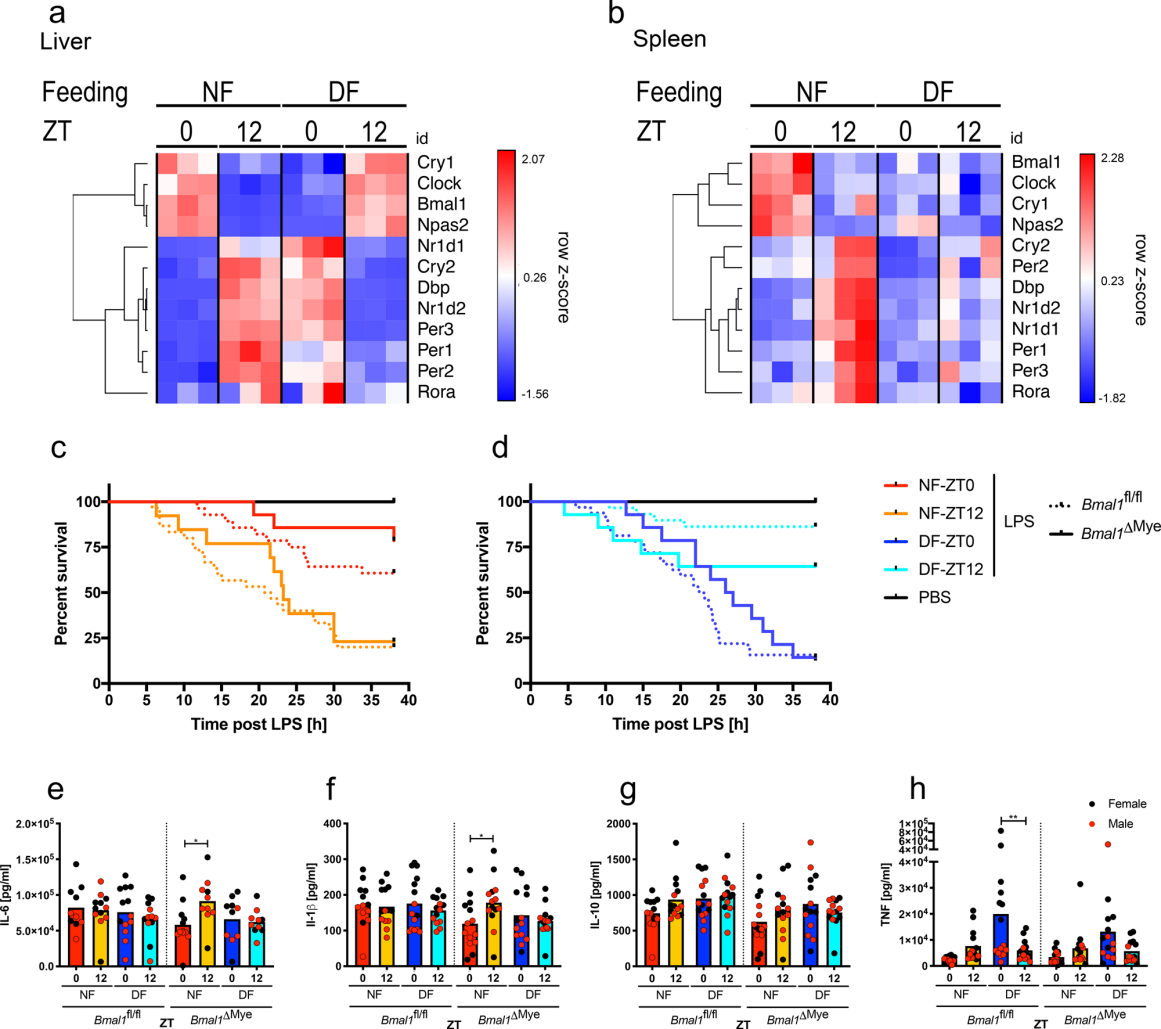
The myeloid clock is dispensable for feeding time-dependent mortality. **a**, **b** Liver (**a**) and spleen (**b**) gene expression profile of selected clock genes measured by NanoString on day 5 of TRF at indicated times in female mice. Unsupervised gene clustering. **c**, **d** Morbidity in night-time (**c**) or day-time (**d**) fed myeloid cell specific *Bmal1*^ΔMye^ (solid) (NF ZT0 vs. 12 *p* = 0.0032, DF ZT 0 vs. 12 ns, ZT0 NF vs. DF *p* = 0.0005, ZT12 NF vs. DF ns), or control *Bmal1*^fl/fl^ (dashed line) mice (NF 0 vs. 12 *p* = 0.0008, DF 0 vs. 12 *p* < 0.0001, ZT0 NF vs. DF *p* < 0.0001, ZT12 NF vs. DF *p* < 0.0001) following 20 mg/kg LPS i.p. on day 5 of TRF at ZT0 or ZT12 as indicated (*Bmal1*^ΔMye^ vs. respective *Bmal1*^fl/fl^ all ns). Statistical analysis according to Mantel–Cox. Experimental numbers: *Bmal1*^ΔMye^: DF-ZT0-LPS *N*_female_ = 7 *N*_male_ = 7, NF-ZT0-LPS *N*_female_ = 7 *N*_male_ = 7, DF-ZT12-LPS *N*_female_ = 7 *N*_male_ = 7, NF-ZT12-LPS *N*_female_ = 6 *N*_male_ = 7, DF-ZT0-PBS *N*_female_ = 2 *N*_male_ = 4, NF-ZT0-PBS *N*_female_ = 2 *N*_male_ = 3, DF-ZT12-PBS *N*_female_ = 1 *N*_male_ = 5, NF-ZT12-PBS *N*_female_ = 1 *N*_male_ = 4. *Bmal1*^fl/fl^: DF-ZT0-LPS *N*_female_ = 16 *N*_male_ = 16, NF-ZT0-LPS *N*_female_ = 16 *N*_male_ = 12, DF-ZT12-LPS *N*_female_ = 15 *N*_male_ = 14, NF-ZT12-LPS *N*_female_ = 16 *N*_male_ = 14, DF-ZT0-PBS *N*_female_ = 8 *N*_male_ = 4, NF-ZT0-PBS *N*_female_ = 8 *N*_male_ = 5, DF-ZT12-PBS *N*_female_ = 7 *N*_male_ = 5, NF-ZT12-PBS *N*_female_ = 5 *N*_male_ = 6. **e**–**h** Serum cytokines in *Bmal1*^ΔMye^ and *Bmal1*^fl/fl^ control mice 2 h post LPS stimulation (20 mg/kg) on day 5 of TRF at ZT0 or ZT12 as indicated. Female in black, male in red. Box indicates mean. Statistical analysis according to two-way ANOVA (within genotype) and Sidak’s multiple comparison (ZT0 vs. ZT12) as indicated. **e** *Bmal1*^fl/fl^: ns. *Bmal1*^ΔMye^: Interaction *F*_(1,38)_ = 4.234, *p* = 0.0465, ZT *F*_(1,38)_ = 2.668, ns, Feeding *F*_(1,38)_ = 1.424, ns. *Bmal1*^fl/fl^: NF-ZT0 *N*_female_ = 6 *N*_male_ = 4, NF-ZT12 *N*_female_ = 6 *N*_male_ = 6, DF-ZT0 *N*_female_ = 7 *N*_male_ = 6, DF-ZT12 *N*_female_ = 7 *N*_male_ = 6. *Bmal1*^ΔMye^: NF-ZT0 *N*_female_ = 6 *N*_male_ = 6, NF^-^ZT12 *N*_female_ = 3 *N*_male_ = 7, DF-ZT0 *N*_female_ = 6 N_male_ = 4, DF-ZT12 *N*_female_ = 4 *N*_male_ = 6. *p = 0.0221. **f** *Bmal1*^fl/fl^: ns, *Bmal1*^ΔMye^: Interaction *F*_(1,52)_ = 4.574, *p* = 0.0372, ZT *F*_(1,52)_ = 1.441, ns, Feeding *F*_(1,52)_ = 0.6317, ns. *p = 0.0435. **g** ns. **h** *Bmal1*^fl/fl^: Interaction *F*_(1,52)_ = 8.776, *p* = 0.0046, ZT *F*_(1,52)_ = 1.923, ns, Feeding *F*_(1,52)_ = 5.985, *p* = 0.0.0178. *Bmal1*^ΔMye^: Interaction *F*_(1,52)_ = 6.532, *p* = 0.0136, ZT *F*_(1,52)_ = 0.9279, ns, Feeding *F*_(1,52)_ = 4.214, *p* = 0.0.451. **p = 0.0057. **f**–**h** *Bmal1*^fl/fl^: NF-ZT0 *N*_female_ = 7 *N*_male_ = 6, NF-ZT12 *N*_female_ = 8 *N*_male_ = 6, DF-ZT0 *N*_female_ = 7 *N*_male_ = 8, DF-ZT12 *N*_female_ = 7 *N*_male_ = 7. *Bmal1*^ΔMye^: *N*_female_ = 7 *N*_male_ = 7.

To better understand the influence of food and light cycle on the circadian clock and downstream metabolic and inflammatory outputs, we measured expression of key circadian clock components in the liver and spleen in NF or DF mice (Fig. 3a, b). In the liver, time-of-day-dependent hepatic expression of many clock genes and selected metabolic and inflammatory regulators were clearly reversed at ZT0 and ZT12 in DF animals. In contrast, DF merely dampened the patterns of gene expression in spleen, in macrophage-enriched peritoneal exudate cells, and adipocytes at the selected time-points on day 5 of TRF, indicating greater liver transcriptional sensitivity to feeding cues (Supplementary Fig. 2c–f).

### Myeloid BMAL1 is not required for feeding cycle-dependent LPS mortality

Macrophages play an important role in sensing innate immune stimuli and previous reports linked diurnal changes in the inflammatory response to the myeloid compartment^4,6,24^. However, in concordance with no clear transcriptional adaptation to reversed feeding cycles in peritoneal macrophages, deletion of BMAL1 in the myeloid lineage did not alter the food-regulated sensitivity to LPS, as reversal of the feeding cycle in LysM^Cre^ × BMAL1^loxP/loxP^ (*Bmal1*^ΔMye^) mice reversed the peak sensitivity to LPS from ZT12 to ZT0 (Fig. 3c, d), and levels of serum cytokines were comparable in *Bmal1*^ΔMye^ vs. *Bmal1*^fl/fl^ animals (Fig. 3e–h). Although this result contrasts with previous reports of enhanced sensitivity to LPS in myeloid cell-specific BMAL1 deficiency^4,5^, our findings are in a model with earlier onset of symptoms, and are supported by a recent study^25^, which also challenges the notion of enhanced mortality in these conditional knockout mice.

### Daily changes in LPS sensitivity depends on BMAL1 in hepatocytes

**Fig. 4:**
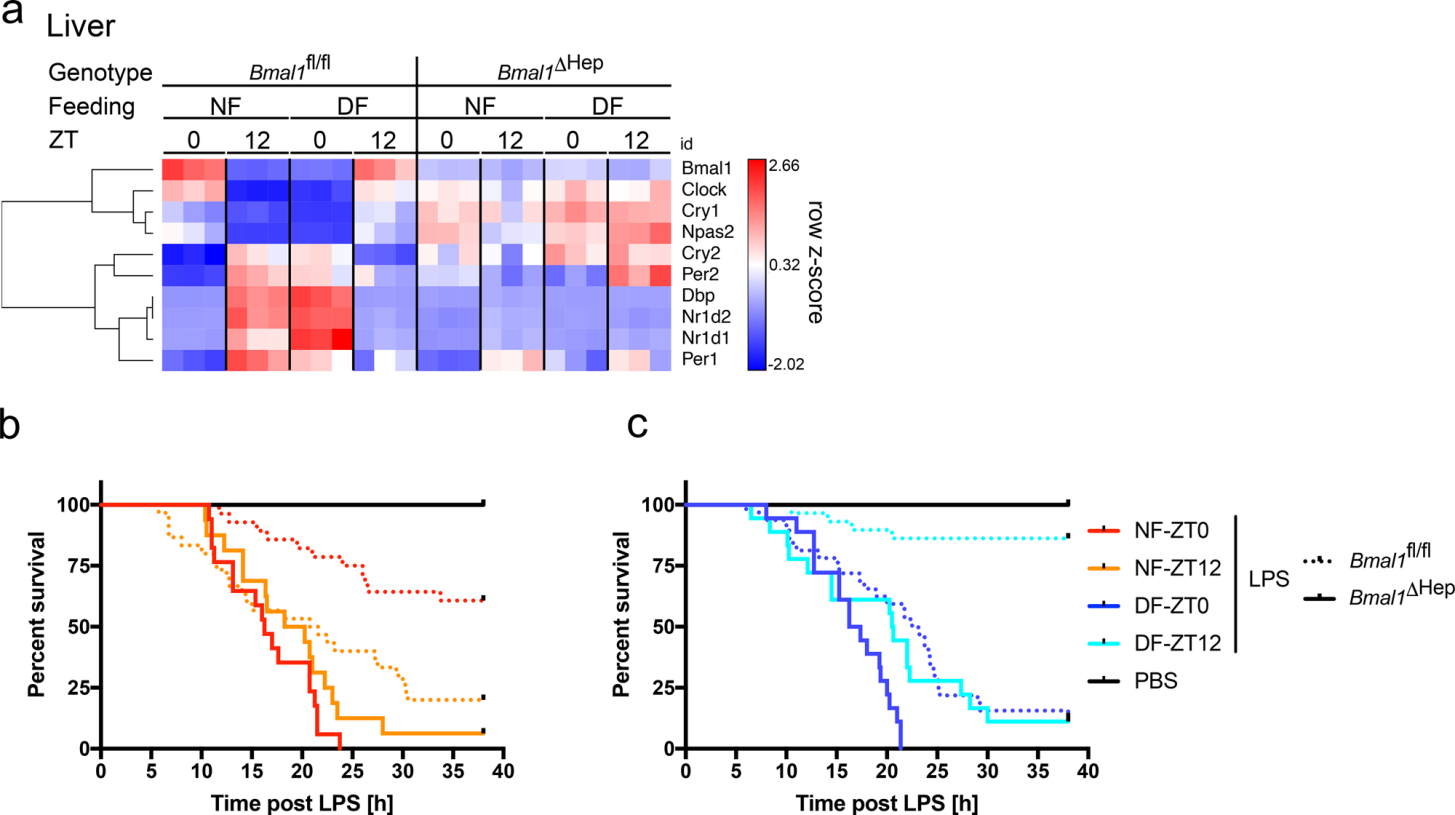
The liver clock regulates susceptibility to LPS. **a** Gene expression profile of selected clock genes in liver measured by NanoString in female *Bmal1*^fl/fl^ or *Bmal1*^ΔHep^ mice on day 5 of TRF at the indicated times. Unsupervised gene clustering. *N* = 3. **b**, **c** Morbidity in night-time (**b**) or day-time (**c**) *Bmal1*^ΔHep^ solid line (NF 0 vs. 12 ns, DF 0 vs. 12 *p* = 0.0163, ZT0 NF vs. DF ns, ZT12 NF vs. DF ns) or control *Bmal1*-floxed (dashed) mice (NF 0 vs. 12 *p* = 0.0008, DF 0 vs. 12 *p* < 0.0001, ZT0 NF vs. DF *p* < 0.0001, ZT12 NF vs. DF *p* < 0.0001), following 20 mg/kg LPS i.p. on day 5 of TRF at ZT0 or ZT12 as indicated. Statistical analysis according to Mantel–Cox. Experimental numbers: *Bmal1*^ΔHep^ mice: DF-ZT0-LPS *N*_female_ = 9 *N*_male_ = 9, NF-ZT0-LPS *N*_female_ = 9 *N*_male_ = 8, DF-ZT12-LPS *N*_female_ = 9 *N*_male_ = 9, NF-ZT12-LPS *N*_female_ = 8 *N*_male_ = 8, DF-ZT0-PBS *N*_female_ = 3 *N*_male_ = 3, NF-ZT0-PBS *N*_female_ = 2 *N*_male_ = 4, DF-ZT12-PBS *N*_female_ = 3 *N*_male_ = 3, NF-ZT12-PBS *N*_female_ = 2 *N*_male_ = 4. *Bmal1*^fl/fl^: DF-ZT0-LPS *N*_female_ = 16 *N*_male_ = 16, NF-ZT0-LPS *N*_female_ = 16 *N*_male_ = 12, DF-ZT12-LPS *N*_female_ = 15 *N*_male_ = 14, NF-ZT12-LPS *N*_female_ = 16 *N*_male_ = 14, DF-ZT0-PBS *N*_female_ = 8 *N*_male_ = 4, NF-ZT0-PBS *N*_female_ = 8 *N*_male_ = 5, DF-ZT12-PBS *N*_female_ = 7 *N*_male_ = 5, NF-ZT12-PBS *N*_female_ = 5 *N*_male_ = 6.

**Fig. 5:**
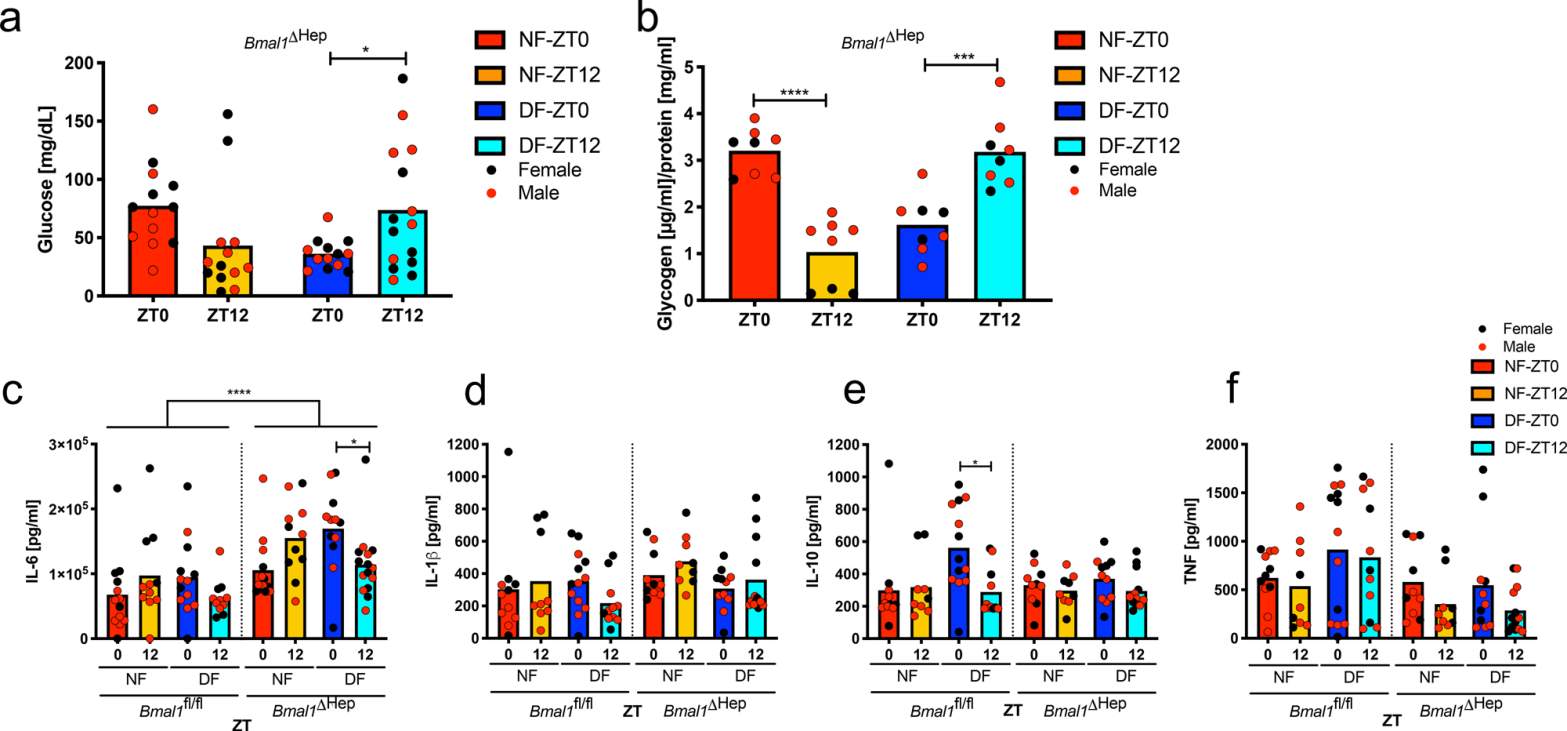
Feeding-related glucose metabolism is liver-clock independent. **a** Serum glucose 6 h post LPS (20 mg/kg) stimulation at ZT0 or ZT12 as indicated on day 5 of TRF in *Bmal1*^ΔHep^. Female in black, male in red. NF-ZT0 *N*_female_ = 6 *N*_male_ = 7, NF-ZT12 *N*_female_ = 6 *N*_male_ = 7, DF-ZT0 *N*_female_ = 6 *N*_male_ = 7, DF-ZT12 *N*_female_ = 8 *N*_male_ = 7. Box indicates mean. Statistical analysis according to two-way ANOVA and Sidak’s multiple comparison (ZT0 vs. ZT12) as indicated. Interaction *F*_(1,50)_ = 10.2, *p* = 0.0024, ZT *F*_(1,50)_ = 0.02174, ns, Feeding *F*_(1,50)_ = 0.2325, ns. *p = 0.0395. **b** Baseline liver glycogen content on day 5 of TRF at ZT0 or ZT12 in *Bmal1*^ΔHep^. Female in black, male in red. *N*_female_ = 3 *N*_male_ = 5. Box indicates mean. Statistical analysis according to two-way ANOVA and Sidak’s multiple comparison (ZT0 vs. ZT12) as indicated. Interaction *F*_(1,28)_ = 64.45, *p* < 0.0001, ZT *F*_(1,28)_ = 1.682, ns, Feeding *F*_(1,28)_ = 1.431, ns. ***p = 0.0001 ****p < 0.0001. **c**–**f** Serum cytokines in *Bmal1*^ΔHep^ or *Bmal1*^fl/fl^ 6 h post LPS stimulation (20 mg/kg) after 5 d of TRF at ZT0 or ZT12 as indicated. Box indicates mean. Female in black, male in red. Statistical analysis according to two-way ANOVA (within genotype) and Sidak’s multiple comparison (ZT0 vs. ZT12) as indicated. **c** *Bmal1*^fl/fl^: ns. *Bmal1*^ΔHep^: Interaction *F*_(1_^,^_47)_ = 11.12, *p* = 0.0017, ZT *F*_(1,47)_ = 0.03561, ns, Feeding *F*_(1,47)_ = 0.5365, ns. p* = 0.027. *p* < 0.0001 *Bmal1*^fl/fl^ vs. *Bmal1*^ΔHep^ according to Mann–Whitney. *Bmal1*^ΔHep^: NF-ZT0 *N*_female_ = 6 *N*_male_ = 7, NF^-^ZT12 *N*_female_ = 6 *N*_male_ = 5, DF-ZT0 *N*_female_ = 6 *N*_male_ = 6, DF-ZT12 *N*_female_ = 8 *N*_male_ = 7. *Bmal1*^fl/fl^: NF-ZT0 *N*_female_ = 5 *N*_male_ = 7, NF-ZT12 *N*_female_ = 5 *N*_male_ = 7, DF-ZT0 *N*_female_ = 6 *N*_male_ = 7, DF-ZT12 *N*_female_ = 4 *N*_male_ = 7. **d** ns. **e** *Bmal1*^fl/fl^: Interaction *F*_(1,40)_ = 4.435, *p* = 0.0415, ZT *F*_(1,40)_ = 3.31, ns, Feeding *F*_(1,40)_ = 2.803, ns. *Bmal1*^ΔHep^ ns. p* = 0.0116. **f** *Bmal1*^fl/fl:^ ns. *Bmal1*^ΔHep^: Interaction *F*_(1,39)_ = 0.01987, ns, ZT *F*_(1,39)_ = 4.335, *p* = 0.0439, Feeding *F*_(1,39)_ = 01707, ns. **d**–**f** *Bmal1*^ΔHep^: NF-ZT0 *N*_female_ = 5 *N*_male_ = 5, NF^-^ZT12 *N*_female_ = 4 *N*_male_ = 5, DF-ZT0 *N*_female_ = 5 *N*_male_ = 6, DF-ZT12 *N*_female_ = 7 *N*_male_ = 6. *Bmal1*^fl/fl^: NF-ZT0 *N*_female_ = 5 *N*_male_ = 6, NF-ZT12 *N*_female_ = 3 *N*_male_ = 6, DF-ZT0 *N*_female_ = 6 *N*_male_ = 7, DF-ZT12 *N*_female_ = 4 *N*_male_ = 7.

Given the importance of the liver in metabolism and dependence of clock gene expression on the feeding cycle, we deleted hepatocyte BMAL1 in Albumin^Cre^ × BMAL1^loxP/loxP^ (*Bmal1*^ΔHep^) mice, which effectively disrupted time-of-day-dependent gene expression of BMAL1 and core hepatic clock genes (Fig. 4a). Remarkably, these mice entirely lost food and time dependency in their susceptibility to LPS, with >90% of mice in all groups succumbing to LPS (Fig. 4b, c). Deletion of BMAL1 in hepatocytes did not alter baseline glucose levels (Supplementary Fig. 3a), the hypoglycaemia in NF mice challenged with LPS at ZT12 and DF mice at ZT0 (Fig. 5a), or glycogen stores in the fed vs. fasted state (Fig. 5b). Gluconeogenesis, as measured by pyruvate tolerance test (PTT) (Supplementary Fig. 3b), as well as liver expression of *Pepck* and *Pygl* (Supplementary Fig. 3c, d), and a selection of metabolic markers in the serum of *Bmal1*^ΔHep^ mice also mirrored *Bmal1*^fl/fl^ values under similar feeding conditions (Supplementary Fig. 4a–g). *Bmal1*^ΔHep^ mice further had no spontaneous phenotype as reflected by appearance, lean and fat mass (as defined by EchoMRI), RER or food intake (Supplementary Fig. 5a–c). Measurement of serum cytokines in *Bmal1*^ΔHep^ mice revealed that levels of IL-6, which is predominantly synthesized by hepatocytes after LPS^26^, were significantly higher in *Bmal1*^ΔHep^ mice after LPS challenge compared to *Bmal1*^fl/fl^ (Fig. 5c) or *Bmal1*^ΔMye^ (Fig. 3e) mice, while other cytokines as well as soluble CD14 at baseline and post LPS mirrored *Bmal1*^fl/fl^ levels (Fig. 5d–f, Supplementary Fig. 5d, e). These data suggest that BMAL1 in hepatocytes may contribute to an attenuation of the inflammatory response, at least in part by dampening IL-6 production. The mechanism and biological consequences of this elevated, yet feeding-time-dependent IL-6 response in the Bmal1^ΔHep^ mice warrants further investigation.

### RNAseq correlates hepatic FXR signalling with BMAL1-dependent sepsis mortality

**Fig. 6:**
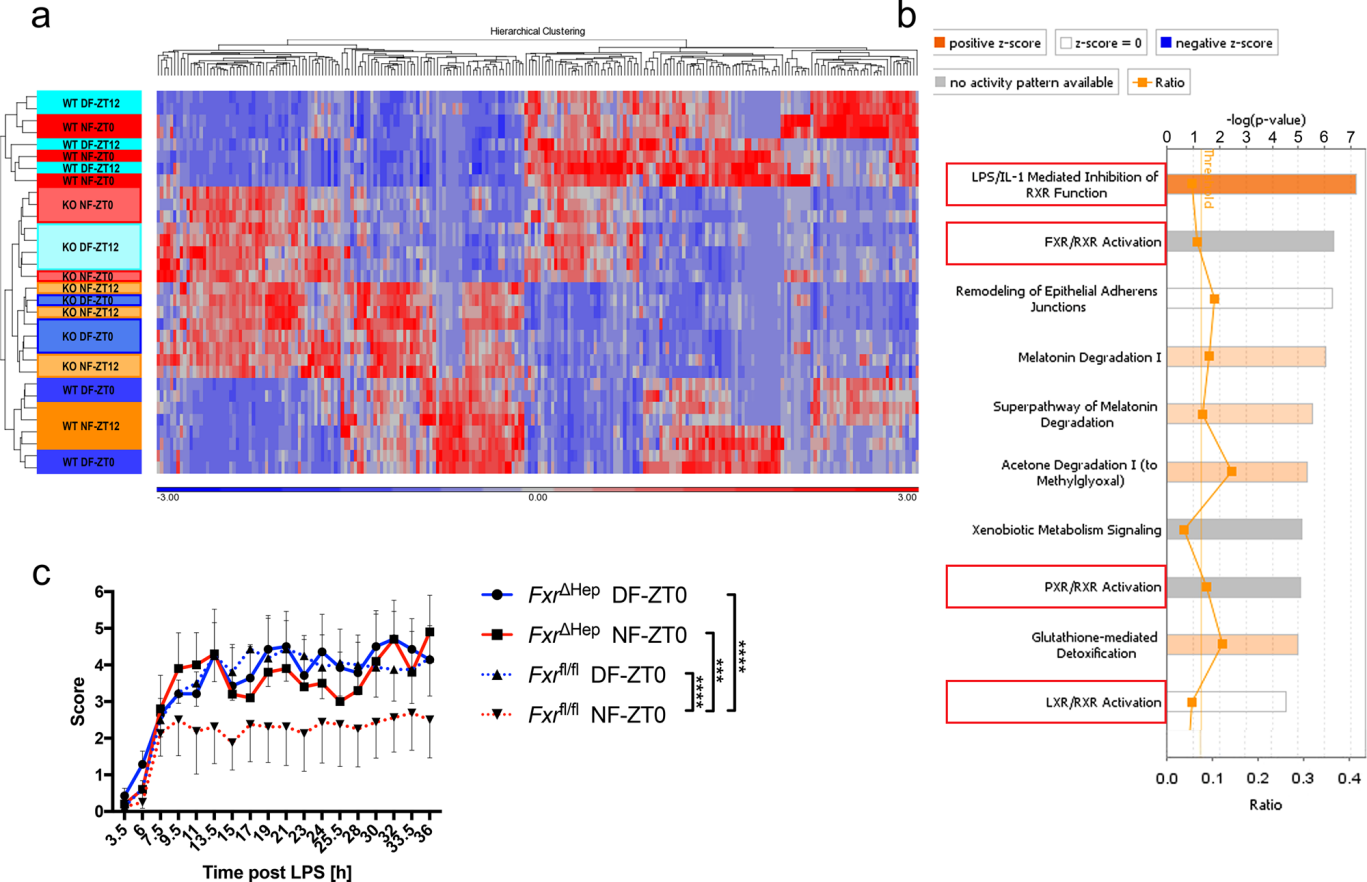
Hepatic FXR regulates early susceptibility to LPS. **a** One-way ANOVA comparison performed on Log2 transformed RPKM values of *Bmal1*^fl/fl^ and *Bmal1*^ΔHep^ liver on day 5 of TRF at ZT0 or ZT12 as indicated, comparing resistant groups (*Bmal1*^fl/fl^ NF0 and *Bmal1*^fl/fl^ DF12) vs. susceptible groups (*Bmal1*^fl/fl^ NF12, *Bmal1*^fl/fl^ DF0 and all *Bmal1*^ΔHep^ groups). Relative expression for genes that were expressed in at least one animal, a FDR < 0.2, and two-fold changed between resistant vs. susceptible groups (232) are shown in the Heatmap indicating relative expression (RPKM-mean RPKM/standard deviation) for each gene. **b** Core analysis using IPA of genes that were expressed in at least one animal, a FDR < 0.2, and two-fold changed (232) in a one-way ANOVA comparison performed on Log2 transformed RPKM values of *Bmal1*^fl/fl^ and *Bmal1*^ΔHep^ liver on day 5 of TRF at ZT0 or ZT12 as indicated, comparing resistant groups (*Bmal1*^fl/fl^ NF0 and *Bmal1*^fl/fl^ DF12) vs. susceptible groups (*Bmal1*^fl/fl^ NF12, *Bmal1*^fl/fl^ DF0 and all *Bmal1*^ΔHep^ groups). Top 10 canonical pathways are shown. *N* = 4. **c** Clinical score of day-time and night-time fed *Fxr*^ΔHep^ (solid line) or *Fxr*-floxed control female mice (dotted line) following 20 mg/kg LPS i.p. stimulation at ZT0 on day 5 of TRF. Statistical analysis using two-way ANOVA and Sidak’s multiple comparison. *Fxr*^ΔHep^ vs. *Fxr*^fl/fl^ DF: Interaction *F*_(17,234)_ = 0.1507, *p* > 0.9999, time *F*_(17,234)_ = 4.362, *p* < 0.0001, group *F*_(1,234)_ = 0.08568, *p* = 0.77. *Fxr*^ΔHep^ vs. *Fxr*^fl/fl^ NF_:_ Interaction *F*_(17,198)_ = 0^.^1719, *p* > 0.9999, time *F*_(17,198)_ = 1.591, *p* = 0.693, group *F*_(1,198)_ = 12.69, *p* = 0.0005. *Fxr*^ΔHep^ DF vs. NF: Interaction *F*_(17,180)_ = 0.1478, *p* > 0.9999, time *F*_(17,180)_ = 2.754, *p* = 0.0004, group *F*_(1,180)_ = 0.4948, *p* = 0.4827. *Fxr*^fl/fl^ DF vs. NF: Interaction *F*_(17,252)_ = 0.2886, *p* = 0.9978, time *F*_(17,252)_ = 2.514, *p* = 0.0011, group *F*_(1,252)_ = 24.82, *p* < 0.0001. *Fxr*^ΔHep^ DF-LPS *N* = 7, *Fxr*^ΔHep^ NF-LPS *N* = 5, *Fxr*^fl/fl^ DF-LPS *N* = 8, *Fxr*^fl/fl^ NF-LPS *N* = 8. Data are presented as mean values ± SEM.

To better understand the pathways underlying feeding-regulated LPS susceptibility in the presence and absence of BMAL1 and a functional molecular clock in hepatocytes, we performed RNAseq, which revealed tight clustering of gene expression in the 12-h starved (NF-ZT12, DF-ZT0) and fed (NF-ZT0, DF-ZT12) groups in *Bmal1*^fl/fl^ and *Bmal1*^ΔHep^, with the groups protected in the LPS sepsis model (*Bmal1*^fl/fl^ NF-ZT0, DF-ZT12) separated from susceptible groups by the strongest principal component (Supplementary Fig. 6a). More than 80% of 549 genes regulated in the liver by the feeding cycle in *Bmal1*^fl/fl^ mice lost differential expression in *Bmal1*^ΔHep^ animals (Supplementary Table 2, Supplementary Fig. 6b). Pathway analysis of differentially expressed (DE) genes comparing LPS resistant and susceptible *Bmal1*^fl/fl^ groups identified upregulation of farnesoid X receptor (FXR) and retinoid X receptor (RXR) signalling pathways with LPS resistance, whereas these pathways were not feeding-regulated in the *Bmal1*^ΔHep^ liver (Supplementary Fig. 6c, d). Strikingly, when we compared gene expression among all groups of *Bmal1*^fl/fl^ and *Bmal1*^ΔHep^ mice, all BMAL1-deficient groups clustered with LPS sensitive *Bmal1*^fl/fl^ mice (Fig. 6a), with 232 DE genes between *Bmal1*^fl/fl^ LPS-resistant mice and the other LPS susceptible groups (Supplementary Table 3). Pathway analysis of these DE genes revealed similar pathways correlating with LPS resistance, including FXR and RXR signalling (Fig. 6b).

### Hepatic FXR expression regulates feeding-cycle-dependent LPS sensitivity

**Fig. 7:**
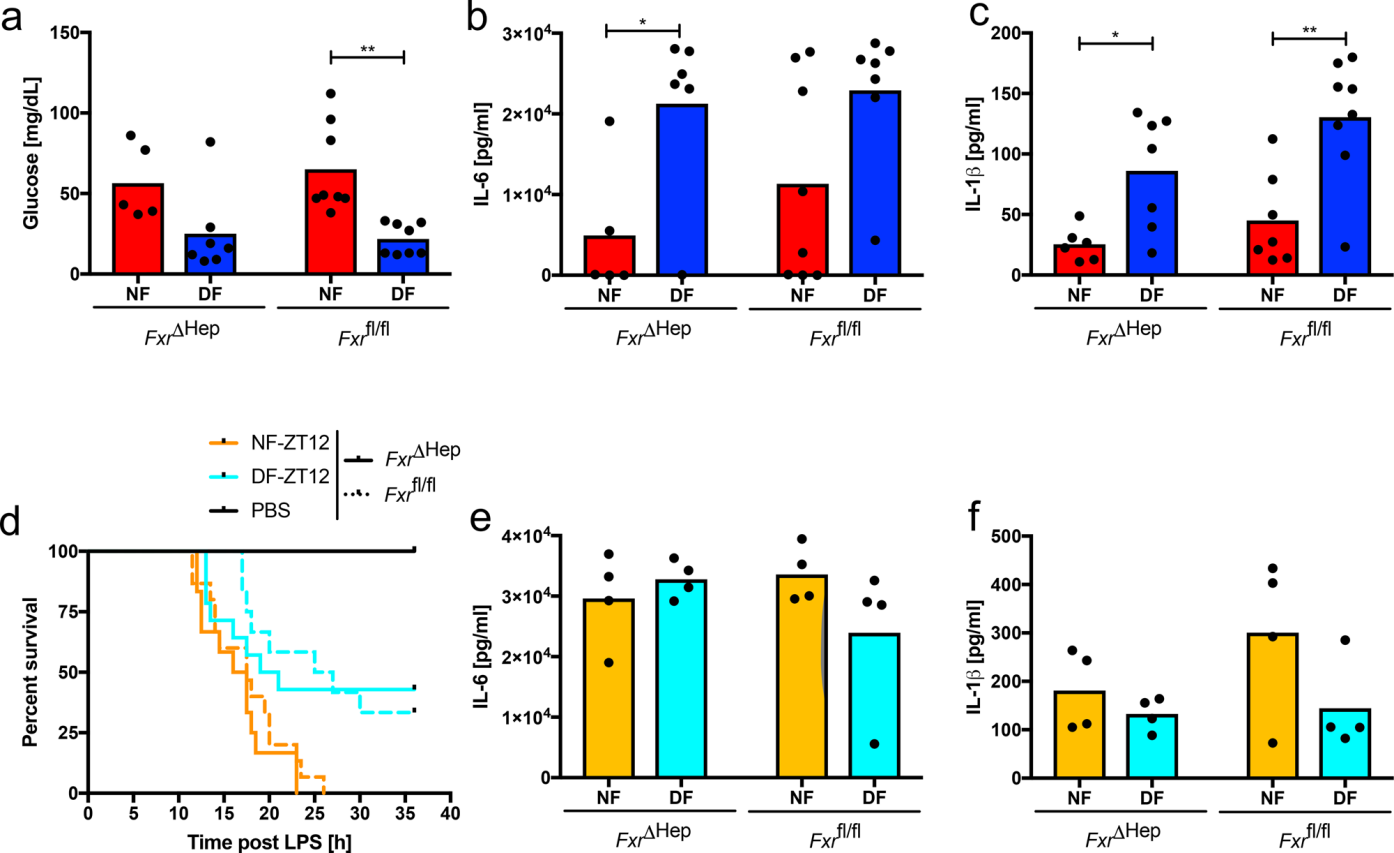
Feeding-related glucose metabolism and cytokine response are unaffected in *Fxr*^ΔHep^ animals. **a** Serum glucose 6 h post 20 mg/kg LPS i.p. at ZT0 on day 5 of TRF in *Fxr*^ΔHep^ and *Fxr*^fl/fl^ control female mice. Statistical analysis using two-way ANOVA and Sidak’s multiple comparison (NF vs. DF) as indicated. Interaction *F*_(1,24)_ = 0.4606, *p* = 0.5038, genotype *F*_(1,24)_ = 0.09389, *p* = 0.7619, feeding *F*_(1,24)_ = 18.28, *p* = 0.0003. **p = 0.0017. *Fxr*^ΔHep^ mice: NF *N* = 5, DF *N* = 7. *Fxr*^fl/fl^: *N* = 8. **b**, **c** IL-6 (E) or IL-1β (F) 6 h post 20 mg/kg LPS i.p. at ZT0 on day 5 of TRF in *Fxr*^ΔHep^ and *Fxr*^fl/fl^ control female mice. Statistical analysis using two-way ANOVA and Sidak’s multiple comparison (NF vs. DF) as indicated. **b** Interaction *F*_(1,24)_ = 0.3339, *p* = 0.5693, genotype *F*_(1,24)_ = 0.9463, *p* = 0.3413, feeding *F*_(1,24)_ = 11.4, *p* = 0.0027. *Fxr*^ΔHep^ mice: NF *N* = 5, DF *N* = 6. *Fxr*^fl/fl^: NF *N* = 8, DF *N* = 7. *p = 0.0027. **c** Interaction *F*_(1,24)_ = 0.6027, *p* = 0.4452, genotype *F*_(1,24)_ = 4.13, *p* = 0.0533, feeding *F*_(1,24)_ = 21.53, *p* = 0.0001. *Fxr*^ΔHep^ mice: NF *N* = 6, DF *N* = 7. *Fxr*^fl/fl^: NF *N* = 7, DF *N* = 8. *p = 0.0286 **p = 0.0011. **d** Morbidity in night-time and day-time fed *Fxr*^ΔHep^ (solid line) (NF vs. DF *p* = 0.021/ns) or *Fxr*^fl/fl^ (dashed) mice (NF vs. DF *p* = 0.0026/*p* = 0.0099), following 35 mg/kg LPS i.p. on day 5 of TRF at ZT12. Statistical analysis according to Mantel–Cox/Gehan–Breslow–Wilcoxon. Experimental numbers: *Fxr*^ΔHep^ mice: DF-LPS *N*_female_ = 11 *N*_male_ = 3, NF-LPS *N*_female_ = 10 *N*_male_ = 2, DF-PBS *N*_female_ = 3 *N*_male_ = 2, NF-PBS *N*_female_ = 1 *N*_male_ = 1. *Fxr*^fl/fl^: DF-LPS *N*_female_ = 9 *N*_male_ = 3, NF-LPS *N*_female_ = 9 *N*_male_ = 6, DF-PBS *N*_female_ = 1, NF-PBS *N*_female_ = 2 *N*_male_ = 1. **e**, **f** IL-6 (**e**) or IL-1β (**f**) 6 h post 35 mg/kg LPS i.p. at ZT12 on day 5 of TRF in *Fxr*^ΔHep^ and *Fxr*^fl/fl^ female mice. No significance using two-way ANOVA. *N* = 4.

We tested the function of the FXR pathway in feeding-cycle regulation of LPS response by hepatocyte-specific disruption of the *Fxr* gene (*Fxr*^ΔHep^). At the same dose of LPS which induced high mortality in C57BL/6J mice, *Fxr*^ΔHep^ and control Cre-negative littermates did not reach disease scores requiring euthanasia. However, using disease severity scores, hepatocyte-specific FXR disruption rendered both night- and DF animals as sensitive to LPS as the susceptible (DF) *Fxr*^fl/fl^ group following ZT0 stimulation (Fig. 6c). Mice in the DF group were equally hypoglycaemic irrespective of FXR signalling status, but in this non-lethal setting, IL-1β and IL-6 levels were higher in the DF groups in both *Fxr*^fl/fl^ and *Fxr*^ΔHep^ mice (Fig. 7a–c), recapitulating previous reports in WT mice^27^ and arguing for a ceiling effect which dampened differential cytokine production at lethal doses of LPS, as seen in Fig. 1e–j. With a higher dose of 35 mg/kg LPS, *Fxr*^ΔHep^ animals in the fed state (DF-ZT12) were still protected from overall mortality, but had increased mortality at timepoints before 20 h compared to *Fxr*^fl/fl^ DF mice following ZT12 stimulation (Fig. 7d). These results demonstrate that at early time-timepoints, FXR deficiency in the liver (FXR^ΔHep^, WT 12h-food deprived), partially overcomes the protective effect of feeding on LPS sepsis. This is supported by Gehan-Breslow-Wilcoxon statistical analysis, which is more sensitive to early events. Using this method, FXR^ΔHep^ mice no longer show a statistical difference in survival between DF and NF groups. In agreement with the constitutively high serum cytokine levels noted after LPS administration regardless of mortality rates in C57BL/6J mice (Figs. 1e–j, 2k–n), serum cytokines were comparably high in *Fxr*^ΔHep^ and control mice (Fig. 7e, f). These results support a role for FXR in protecting mice from LPS-induced lethal sepsis as they transition out of the feeding period. Given the importance of FXR signalling in promoting glucose utilization^28^, hepatic FXR deficiency may allow euglycemic mice in the fed state to phenocopy hypoglycaemic counterparts. At later time points, where the mortality of DF *Fxr*^ΔHep^ converged towards DF *Fxr*^fl/fl^ levels (Fig. 7d), compensatory pathways may become more critical. RNAseq analysis also identified the LXR pathway as feeding and BMAL1 dependent in the mouse liver (Fig. 6b). Given the interplay between the FXR and LXR receptors, and enhanced glucose utilization upon LXR activation^29,30^, both pathways may contribute to protective glucose metabolism and survival in mice with available glycogen stores. Susceptibility of FXR/LXR double-deficient animals in the time-restricted-feeding model, remains to be determined and will aid in unravelling the exact molecular mechanism.

## Discussion

In summary, these findings point to BMAL1 in hepatocytes as a key transducer of nutritional cues into outputs which modulate susceptibility to the lethal effects of LPS. Enhanced susceptibility was further associated with more severe hypoglycaemia rather than enhanced serum cytokines. We have identified the FXR/RXR pathway as a feeding-cycle and BMAL1-dependent pathway and a possible regulator of LPS susceptibility, and confirmed this with functional studies in hepatocyte-specific FXR knockout animals. While LPS stimulation is commonly used to induce TLR4-mediated immune responses and is one model for bacterial sepsis, the translational aspects of these findings in more complex, clinically relevant models, such as CLP, will be of particular interest in future studies, and will allow to dissociate the role for bacterial resistance and tolerance. Given the close relation of food intake and time-of-day under homoeostatic conditions (Supplementary Fig. 1b AL group), our findings are of direct pertinence to the previously described time-of-day-dependent changes in CLP-induced sepsis lethality^17^. The relevance of timed feeding for activation of various immune pathways and the role of food composition in humans will require further investigation and may identify targets to enhance endotoxemia resistance and improve the currently poor survival in severe sepsis.

One of the characteristics of a circadian response is sustained cycling after entrainment. We cannot rule out the possibility that LPS susceptibility depends only on the antecedent feeding state or liver *Bmal1*, rather than being entrained in a circadian manner. However, previous studies with a single 48-hour food deprivation showed reduced hepatic injury and cytokine release in response to LPS^10^, arguing that reversal of a 12-hour feeding cycle has divergent effects on inflammation compared to more prolonged starvation. Our data rather demonstrates, that while feeding dependent availability of glucose and glycogen is a prerequisite, optimal protection from sepsis mortality requires the presence of BMAL1 regulated FXR. In support of this, ad libitum fed animals generally do not phenocopy food deprived animals at any time of the day, but still show differences in mortality at ZT0 vs. ZT12^1,4,31^. Together, these findings change the paradigm of our understanding of diurnal sensitivity to innate immune stimuli and provide new pathways connecting nutritional intake to host defense and adaptive responsiveness to LPS.

## Methods

### Animals and in vivo procedures

All animal protocols were in accordance with Institutional Guidelines and approved by the Institutional Animal Care and Use Committees of the NHLBI and NIAMS of the National Institutes of Health.

#### WT C57BL/6J mice

Wild-type (WT) C57BL/6J (stock number 000664, RRID:IMSR_JAX:000664) mice were purchased from the Jackson Laboratory.

#### Bmal1 LysM-Cre colony

Bmal1 floxed animals (B6.129S4(Cg)*Arntl*^tm1Weit^/J stock number 007668, RRID:IMSR_JAX:007668) and LysM-Cre mice (B6.129P2-*Lyz2*^tm1(cre)Ifo^/J stock number 004781, RRID:IMSR_JAX:004781) were purchased from the Jackson laboratory on C57bl6/J background and have been intercrossed to generate *Bmal1*^fl/fl^LysM-Cre^+/+^, which lack the *Bmal1* gene in the myeloid lineage, and *Bmal1*^fl/fl^LysM-Cre^−/−^ that served as control mice with an intact *Bmal1* gene in all cell types.

#### Bmal1 Alb-Cre colony

Bmal1-floxed animals (B6.129S4(Cg)Arntl^tm1Weit^/J stock number 007668) and Albumin-Cre mice (B6.Cg-^Tg(Alb-cre)21Mgn^/J stock number 003574) have been purchased from the Jackson laboratory on C57BL/6J background and have been intercrossed to generate *Bmal1*^fl/fl^Alb-Cre^+/+^, which lack the *Bmal1* gene in hepatocytes, and *Bmal1*^fl/fl^Alb-Cre^−/−^ that served as control mice with an intact *Bmal1* gene in all cell types.

#### Fxr Alb-Cre colony

Conditional Fxr knockout and control animals were generated and provided by F. J. Gonzalez^32^. Animals were maintained on a C57BL/6J;129S1 background.

#### General animal holding

Animals at 9–13 weeks of age were housed in 12 h:12 h light:dark conditions (~70 Lx at times of illumination) at least one week prior to use, in rooms accessible under red lights during the dark period. Experiments were mainly conducted using male and female mice. No difference was apparent between sexes (red vs. black data points). If not indicated otherwise, only female mice were used.

#### Time restricted feeding (TRF)

Mice were housed in a 12:12 light:dark environment on ad libitum feeding for at least one week, before food was restricted to either the light or the dark phase (Damiola et al., 2000) for 4 days if not specified otherwise. On day 5, mice were either stimulated or killed for tissue collection at ZT0 or ZT12.

#### LPS mortality

Mice were acclimatized to the TRF schedule for 4 days. 24 h before the subjects received an intra peritoneal injection of 20 mg/kg LPS (Sigma, Cat# L2630 Lot# 014M4018V) (sonicated for 5 min prior to preparation in sterile PBS), serum was collected via the submandibular route for baseline level determination. Following stimulation, mice received ad libitum feeding if not indicated otherwise. 2 or 6 h post injection a second submandibular bleed was performed for serum collection as indicated. Mice were killed upon a cumulative score of 8 or a score of 3 in any category and the time-point of death was recorded. The scoring sheet is outlined in the Supplementary methods. Scoring was performed blinded by multiple investigators.

#### CLAMS, comprehensive lab animal monitoring system

The CLAMS (Columbus Instruments) is an enclosed animal holding system that allows measurement of various metabolic parameters such as oxygen consumption, carbon dioxide production, locomotor activity as measured by beam breaks in all three dimensions, and food consumption. Food access as well as timing and duration of the light phase was regulated and adapted to experimental requirements. 12 mice were single housed and data was acquired from each individual separately. Peak RER values continuously increased, but as activity recordings were not indicative of intense activity, an activator bicarbonate buffering which elevates the RER > 1 (Supplementary Fig. 1a), and given that this increase was present in time-restricted and ad libitum-fed animal, suggests a technical rather than biological cause.

### RNA analysis

#### RNA preparation

Tissue samples were temporarily stored at −20 °C in RNAlater stabilization solution (Ambion, Cat# AM7021) to preserve tissue RNA integrity. The sample was homogenized in a Micro-Bead tube 2.38 mm (MoBio, Cat# 13117-50) containing 1 ml Trizol Reagent using a Precellys 24 and subsequently centrifuged at 12,000 × *g* for 10 min at 4 °C. The clear supernatant (without the fatty layer if present) was transferred to a new vial. RNA was isolated using TRIzol Reagent (Life Technologies, Cat# 15596-018) according to the manufacturer’s instructions. Homogenized samples containing 1 ml TRIzol were incubated for 5 min at RT to allow denaturation of proteins before 0.2 ml chloroform was added. During centrifugation at 12,000 × *g* for 15 min at 4 °C, the RNA containing aqueous phase (top phase) was separated from the DNA at the interphase and protein in the (bottom) organic phase. RNA was precipitated from the aqueous phase during incubation with 0.5 ml isopropanol. Following centrifugation at 12,000 × *g* for 10 min at 4 °C, the supernatant was discarded and the pellet washed with 1 ml 75% ethanol, before the pellet (at 7500 × *g* for 5 min at 4 °C) was air-dried for ~10 min at RT. The purified RNA was then resuspended in an appropriate amount of ultra pure water (10–100 µl) and incubated for 12 min at 57 °C to facilitate complete solubilization of the RNA. In order to remove DNA contaminations, the samples were digested with DNase using Turbo DNA-free Kit (Life Technologies, Cat# AM1907) according to the manufacturer’s instructions. 0.1 volume of DNase buffer was added to the sample together with 1 µl of DNase and incubated for 30 min at 37 °C, before 0.1 volume of inactivation reagent was added. Following a 5 min incubation at RT, the sample was centrifuged at 10,000 × *g* for 1.5 min to pellet the DNase inactivation reagent. The DNase digested RNA was then transferred into a fresh vial and stored at −80 °C until further use.

#### NanoString

NanoString technology was used according to the manufacturer’s instructions. Genes involved in the circadian machinery, metabolism and immune response were measured amongst five housekeeping gene mRNAs (*Decr1*, *Fpgs* or *Eif4a2*, *Hmbs*, *Hprt1*, *Ppib*). RNA (800 ng) was mixed with 8 µl Reporter CodeSet in a total volume of 13 µl, before 2 µl Capture ProbeSet was added and the reaction immediately incubated for ~18 h at 65 °C in a thermal cycler. The hybridized samples were then loaded to the NanoString PrepStation for further processing and data collection in the NanoString Digital Analyzer. Target sequences of the CodeSet can be found in Supplementary Method Table 2.

#### RNA sequencing

RNA-seq libraries of purified liver RNA were prepared using NEBNext Ulta II RNA Library Prep Kit for Illumina (New England BioLabs, Cat# E7770S) and sequenced on Illumina Hiseq 3000 with 50 base single end reads. Illumina BCL sequencing results were demultiplexed and converted to FastQ using b2fastq version 2.17.1.14. Reads were mapped to mm10 using Tophat 2.1.1 and RPKM calculations made using Partek Genomic Suite 7.18.0723. ANOVA comparisons were performed on Log2-transformed RPKM (with a 0.1 offset). Cutoffs were based on Fold Change < 2 or <−2; FDR < 0.2 and an RPKM value in at least one animal of >1. Pearson correlations, heat maps and PCA plots were generated using Partek GS. Ingenuity pathway analysis was used to analyse filtered ANOVA gene lists for potentially relevant pathways. For Canonical Pathway results, the height of the bar represents the −log *p*-value of the enrichment of genes within the pathway. The connected line shows the ratio, which is the number of overlapped genes in the pathway vs. the total number of genes in the pathway. The colour of the bar represents the *z*-score, IPA’s prediction of whether the pathway is induced or suppressed based on fold changes.

For RNA-Seq analysis (Fig. 6 and Supplementary Fig. 5), raw FastQ files and processed RPKM table and ANOVA results have been submitted to NCBI GEO under accession number GSE123909.

#### Magnetic bead separation for F4/80 positive cell enrichment

Macrophages were isolated from the peritoneal lavage by negative magnetic isolation, using the EasySep system. To ensure the shortest possible purification times, Mouse Streptavidin Rapidspheres (StemCell Technologies, Catalogue # 19860) were used in conjunction with a custom selection cocktail. The peritoneal cells were pelleted at 805 g for 5 min at 4 °C and subsequently resuspended in 100 µl PBS in a polystyrene FACS tube. Unspecific binding was prevented using 2.5 µl Fc-block ((Bioxcell Cat# CUS-HB-197-A02) 1.2 mg/ml) for 5 min prior to incubation with biotinylated antibodies directed against B-cell (CD19 (BioLegend, Cat# 115504, RRID:AB_313639) & B220 (BioLegend, Cat# 103204, RRID:AB_312989), 0.5 µg), T-cell (Thy1.2, (BioLegend, Cat# 140313), 1 µg) and erythrocyte (TER119 (BioLegend, Cat# 116204, RRID:AB_313705), 1 µg) surface marker for 10 min. The magnetic Rapidspheres were vortexed 1 min before 1.5 µl were added to the isolation mix for 2.5 min. The reaction was resuspended in 2.5 ml PBS and inserted into a purple EasySep magnet for an additional 2.5 min before the purified macrophage suspension was carefully decanted into a new tube. To ensure homogenous distribution, the reaction was vigorously mixed after every addition of reagents.

### Blood works

#### Serum preparation

Serum was separated from whole blood in Z-gel micro tubes (Sarstedt Cat# 41.1500.005) after a 30 min incubation and centrifugation at 10,0000 × *g* for 5 min. Serum was stored at −20 °C until use.

#### PTT

For the PTT^33^, mice received an intraperitoneal injection of sodium pyruvate (Sigma Aldrich, Cat# P2256) (2 g/kg body weight) together with PBS or LPS (20 mg/kg) at ZT0 on day 5 of day time feeding (following 12 h of fasting). Tail vain blood was collected 0, 30, 60, 90, and 120 min after injection to determine blood glucose using a glucometer (AlphaTRAK Zoetis) and appropriate test strips (AlphaTRAK Zoetis, Cat# ART24506).

#### Glycogen measurement

Liver glycogen content was measured using Glycogen Assay Kit (Sigma, Cat#MAK016) according to the manufacturer’s instructions for the colorimetric detection. In short, 10 mg of liver was homogenized in 100 µl water on ice and boiled for 5 min. Following 5 min at 13,000 × *g*, the supernatant was collected and brought to a final volume of 50 µl with Hydrolysis Buffer. 2 µl of hydrolysis enzyme mix was added, the reaction mixed well and incubated for 30 min at RT. Blanks were set up for each reaction by omitting the hydrolysis enzyme mix. After addition of 50 µl master reaction mix, wells were mixed and incubated for 30 min at RT in the dark. The absorbance was measured at 570 and glycogen content was calculated by linear regression from the standard curve.

#### Glucose measurements

Serum glucose was measured using a commercially available kit according to the manufacturer’s instructions (Abcam, Cat# ab65333). In short, 0.5–1.5 µl serum was diluted with Glucose Assay Buffer to a final volume of 50 µl and mixed in a 96-well flat bottom plate with glucose reaction mix. Following an incubation for 30 min at 37 °C in the dark the OD was measured at 570 nm in a microplate reader. Glucose concentrations were then calculated by linear regression from the standard curve.

#### Cytokine measurement

In vivo cytokines were measured at the indicated time points by terminal blood collection following decapitation or via the submandibular route for survival bleeds. Serum was separated in Z-gel microtubes (Sarstedt Cat# 41.1500.005) after a 30 min incubation and centrifugation at 10,000 × *g* for 5 min. Serum was stored at −20 °C until use. Cytokines were measured using the Bio-Plex system (BioRad) in a multiplex assay according to the manufacturer’s instructions. In short, appropriate magnetic capture beads were washed twice with wash buffer in a magnetic plate washer, before the samples and standards were added to the plate and incubated for 30 min while shaking. Following three washes, the respective biotinylated detection antibodies were added for 30 min while shaking, before the plate was washed three times. Lastly, PE-conjugated Streptavidin was added for 10 min before the magnetic beads were washed three times and resuspended in 125 µl assay buffer. The conjugates were then analysed using a Bio-Plex MagPix Multiplex Reader (BioRad). Serum metabolic markers (Supplementary Fig. S4) were similarly measured using this protocol.

#### CD14 measurement

Soluble CD14 levels were quantified using a commercially available ELISA kit (R&D Cat# MC140) according to manufacturer’s instructions. In short, samples were diluted 1:100 and 1:1000 by serial dilution in calibrator diluent, standards were prepared as instructed in calibrator diluent. Assay diluent was added to the microplate pre-coated in a monoclonal antibody specific to mouse CD14. Standards, diluted samples, and sCD14 controls were then added to the plate and incubated for 2 h while shaking. Plate was washed four times before a polyclonal antibody specific for mouse CD14 conjugated to horseradish peroxidase was added then incubated for 2 h while shaking. Wash steps were repeated, and a substrate solution consisting of equal parts stabilized hydrogen peroxide and stabilized chromogen (tetramethylbenzidine) were added and incubated for 30 min without shaking. A stop solution of hydrochloric acid was added and the plate was read at 450 nm. sCD14 measurements were calculated by plotting against the standard curve and multiplying by the dilution factor.

#### Corticosterone measurement

Corticosterone levels were quantified using a commercially available ELISA kit (Enzo Cat# ADI-900-097). In short, serum samples were incubated with steroid displacement reagent to free bound Corticosterone from steroid binding proteins. 1:40 diluted samples and freshly prepared Corticosterone standards were then loaded to a Donkey anti-Sheep IgG Microtiter Plate and incubated on a plate shaker for 2 h at 453 × *g* RT. Following three washes, pNpp Stop solution was added and absorption was read at 405 nm, while absorption at 580 nm was used for correction. Corticosterone concentrations were extrapolated from a percent bound versus corticosterone standard plot.

#### β-Hydroxybutyrate measurement

β-Hydroxybutyrate was measured using a commercially available colorimetric assay (Cayman, Cat# 700190). Oxidation of β-hydroxybutyrate to acetoacetate results in simultaneous reduction of NAD^+^ to NADH + H^+^. The colorimetric detector WST-1 then reacts with NADH to form a formazan dye, whose absorbance at 450 nm is proportional to the β-hydroxybutyrate concentration. To initiate the reaction, serum samples and β-hydroxybutyrate standards were incubated for 30 min at RT in the dark with Developer solution. Absorbance of β-hydroxybutyrate standards was plotted against the concentrations followed by linear regression, from which sample concentrations were extrapolated.

### Statistics

Error bars represent the standard error of the mean. Statistical analysis was performed using appropriate tests in Prism7. Based on the relatively small sample sizes, non-parametric analysis (Kruskal–Wallis or Mann–Whitney) were used for one-factorial testing. Due to lack of readily available two-factorial non-parametric tests, parametric two-way ANOVA was performed for two-factorial testing. The authors refrained from using higher order statistical analysis (e.g. three-way ANOVA) as the complexity of the results was not proportional to the addition of useful information they would provide. Statistical testing of survival data was performed using Mantel–Cox and where appropriate (due to early/late time-point differences) Gehan–Breslow–Wilcoxon analysis. CLAMS data was analysed using CalR 1.2 (https://calrapp.org)^34^. Data points represent distinct samples in all figures.

## Supplementary information

Supplementary Information

## Data Availability

Source data are provided with this paper, Raw FastQ files and processed RPKM table and ANOVA results have been submitted to NCBI GEO under accession number GSE123909. Source data are provided with this paper.

## Source data

Source Data
