# Supplementary material for: Feeding-induced resistance to acute lethal sepsis is dependent on hepatic BMAL1 and FXR signalling
Source: Nat Commun. 2021 May 12;12:2745. doi: 10.1038/s41467-021-22961-z (PMC8115055; doi:10.1038/s41467-021-22961-z)
Supplement: Supplementary file 1 — Supplementary Information [file 41467_2021_22961_MOESM1_ESM.docx]

**SUPPLEMENTARY DATA**


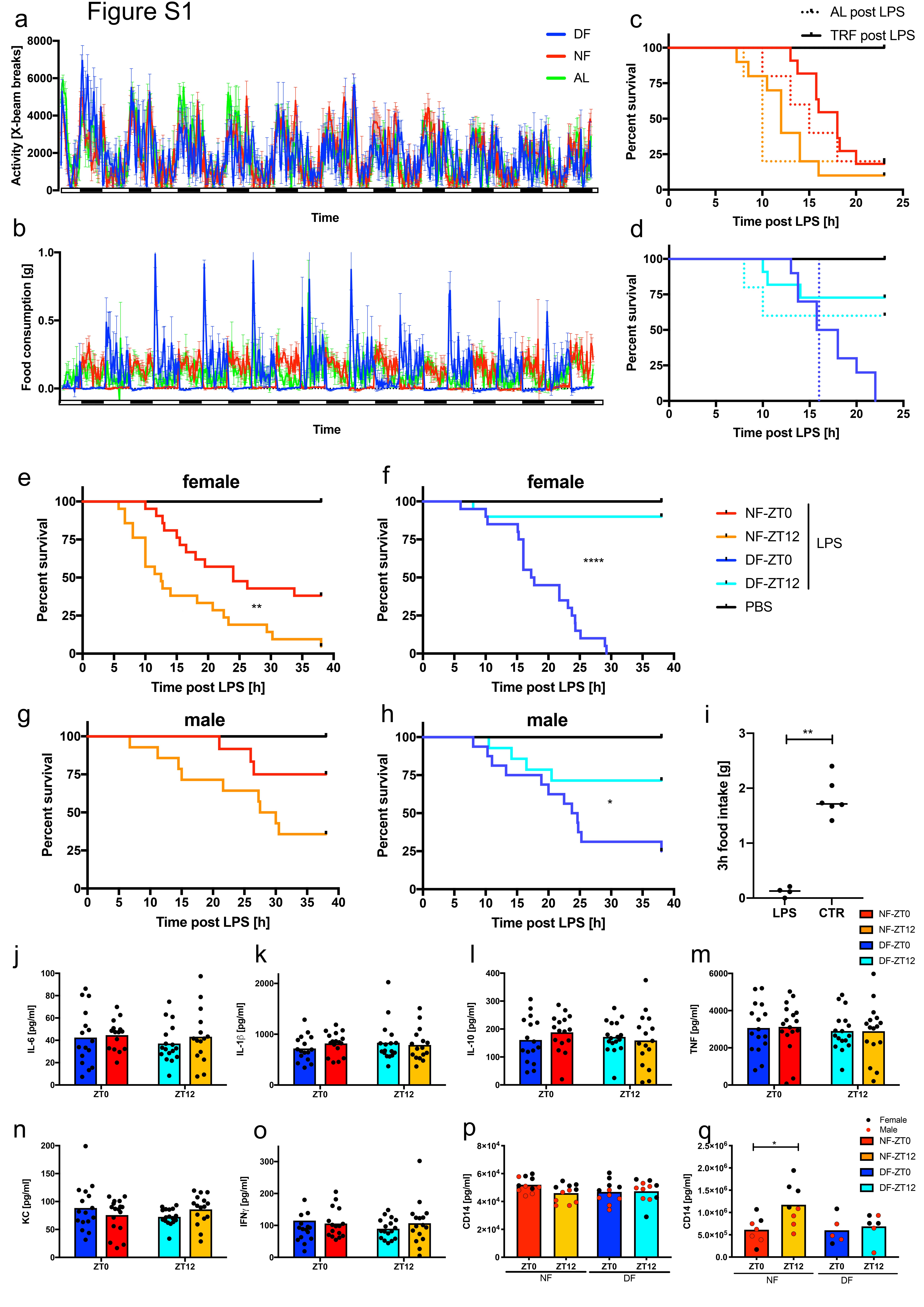


Supplementary Figure 1: **Time restricted feeding sustains overall behavior and baseline serum cytokines.** A, B) Activity (A) and food consumption (B) in female TRF mice over time. Black bars indicate dark phases. Representative of 7 individual experiments. N=4. Data are presented as mean values +/- SEM. Full statistical analysis using CalR in supplementary table 1. A) Activity values min: DF=3, NF=4, AL=4, max: DF=9417, NF=7260, AL=6993. B) Food consumption values max: DF=1.41, NF=1.23, AL=01.16. C, D) Morbidity following 20 mg/kg LPS i.p. on day 5 of TRF at ZT0 or ZT12 as indicated, with solid lines representing continued TRF (NF 0 vs. 12 p=0.0372, DF 0 vs. 12 p=0.0055, ZT0 NF vs. DF ns, ZT12 NF vs. DF p=0.0066), and dotted lines representing *ad libitum* feeding post LPS (NF 0 vs. 12 p=0.4335, DF 0 vs. 12 p=0.4383, ZT0 NF vs. DF p=0.8617 ZT12 NF vs. DF p=0.9844) in female mice. Experimental numbers: Continued TRF DF-ZT0-LPS N=10, NF-ZT0-LPS N=11, DF-ZT12-LPS N=11, NF-ZT12-LPS N=10, PBS N=6, Ad lib following LPS: DF-ZT0-LPS N=4, NF-ZT0-LPS N=5, DF-ZT12-LPS N=5, NF-ZT12-LPS N=5, PBS N=3). Statistical analysis according to Mantel-Cox. E-H) Morbidity in female night-time (p=0.0075) (E) or day-time fed (p<0.0001) (F) or male night-time (p=0.0557) (G) or day-time fed (p=0.0262) (H) C57BL/6J and *Bmal1*^fl/fl^ control mice following 20 mg/kg LPS or PBS i.p. administered at ZT0 or ZT12 on day 5 of TRF with the indicated schedule. Statistical analysis according to Mantel-Cox comparing LPS stimulated groups. *p<0.05, **p<0.01, ****p<0.0001. Experimental numbers: DF-ZT0-LPS N_female_=20 N_male_=16, NF-ZT0-LPS N_female_=21 N_male_=12, DF-ZT12-LPS N_female_=20 N_male_=14, NF-ZT12-LPS N_female_=21 N_male_=14, DF-ZT0-PBS N_female_=11 N_male_=4, NF-ZT0-PBS N_female_=11 N_male_=5, DF-ZT12-PBS N_female_=10 N_male_=5, NF-ZT12-PBS N_female_=8 N_male_=6. I) ZT0-ZT3 3h food intake 24h pre (CTR) or post 20 mg/kg LPS i.p. (LPS) in female TRF mice at ZT0. **p=0.0095. Significance according to Mann-Whitney. J-O) Baseline serum cytokines on day 5 of TRF at ZT0 or ZT12 as indicated. J) NF-ZT0 N=15, NF-ZT12 N=15, DF-ZT0 N=16, DF-ZT12 N=17. K) NF-ZT0 N=17, NF-ZT12 N=16, DF-ZT0 N=16, DF-ZT12 N=17. L) NF-ZT0 N=16, NF-ZT12 N=16, DF-ZT0 N=16, DF-ZT12 N=17 M) NF-ZT0 N=17, NF-ZT12 N=16, DF-ZT0 N=16, DF-ZT12 N=17. N) NF-ZT0 N=17, NF-ZT12 N=16, DF-ZT0 N=16, DF-ZT12 N=17, O) NF-ZT0 N=16, NF-ZT12 N=16, DF-ZT0 N=16, DF-ZT12 N=17. Females only. Box indicates mean. No statistical significance according to two-way ANOVA. P) Baseline serum soluble CD14 on day 5 of TRF at ZT0 or ZT12 as indicated. Box indicates mean. Female in black, male in red. N_female_=5 N_male_=6. No statistical significance using two-way ANOVA. Q) Serum soluble CD14 6h post LPS stimulation (20 mg/kg) on day 5 of TRF at ZT0 or ZT12 as indicated. NF-ZT0 N_female_=3 N_male_=4, NF-ZT12 N_female_=4 N_male_=4, DF-ZT0 N_female_=2 N_male_=3, DF-ZT12 N_female_=3 N_male_=3. Box indicates mean. Female in black, male in red. Two-way ANOVA and Sidak’s multiple comparison as indicated. Interaction F_(1,22)_=2.634, ns, ZT F_(1,22)_=4.944, p=0.0368, feeding F_(1,22)_=2.913, ns. *p=0.0426.


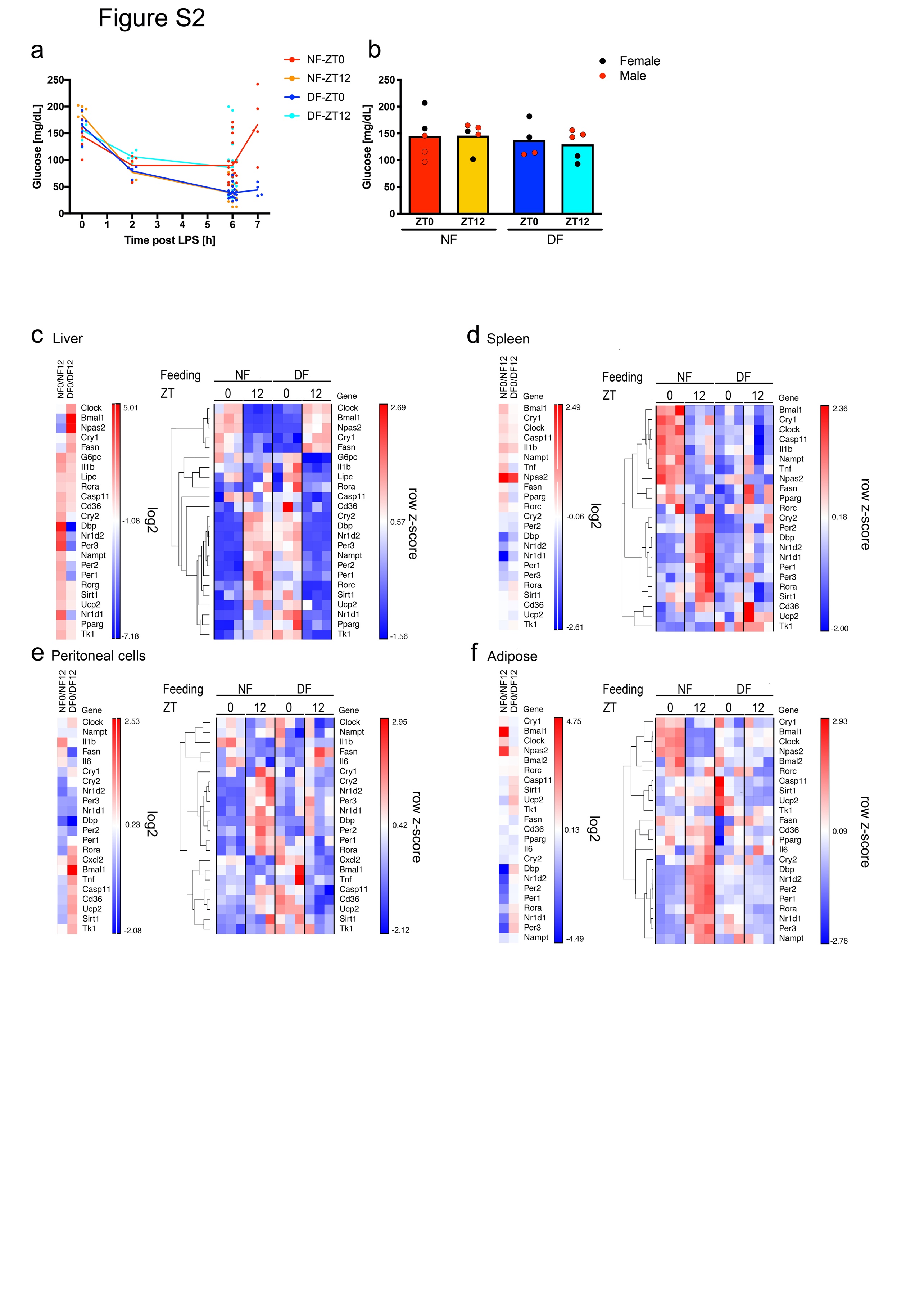


Supplementary Figure 2: **Time restricted feeding resets RNA expression exceptionally in the liver.** A) Serum glucose levels over time following LPS stimulation (20mg/kg) on day 5 of TRF at ZT0 or ZT12 as indicated. Two-way ANOVA NF0-DF0 F_(3, 58)_=10.8 p<0.0001, NF12-DF12 F_(2, 50)_=5.159 p=0.0092. NF-ZT0 (t0) N=6, NF-ZT0 (t2) N=5, NF-ZT0 (t6) N=18, NF-ZT0 (t7) N=5, NF-ZT12 (t0) N=7, NF-ZT12 (t2) N=5, NF-ZT12 (t6) N=16, DF-ZT0 (t0) N=6, DF-ZT0 (t2) N=4, DF-ZT0 (t6) N=18, DF-ZT0 (t7) N=4, DF-ZT12 (t0) N=5, DF-ZT12 (t2) N=5, DF-ZT12 (t6) N=18. Line indicates mean. B) Baseline serum glucose on day 5 of TRF at ZT0 or ZT12 as indicated. NF-ZT0 N_female_=2 N_male_=3, NF-ZT12 N_female_=2 N_male_=3, DF-ZT0 N_female_=2 N_male_=2, DF-ZT12 N_female_=2 N_male_=3. Box indicates mean. Female in black, male in red. No statistical significance according to two-way ANOVA. C-F) NanoString RNA expression profile in selected tissues as indicated, extracted on day 5 of TRF at ZT0 or ZT12 from female mice. N=3. Left: average ZT0/ZT12 expression ratios in night- or day-time fed animals. Right: unsupervised gene clustering, row normalized.


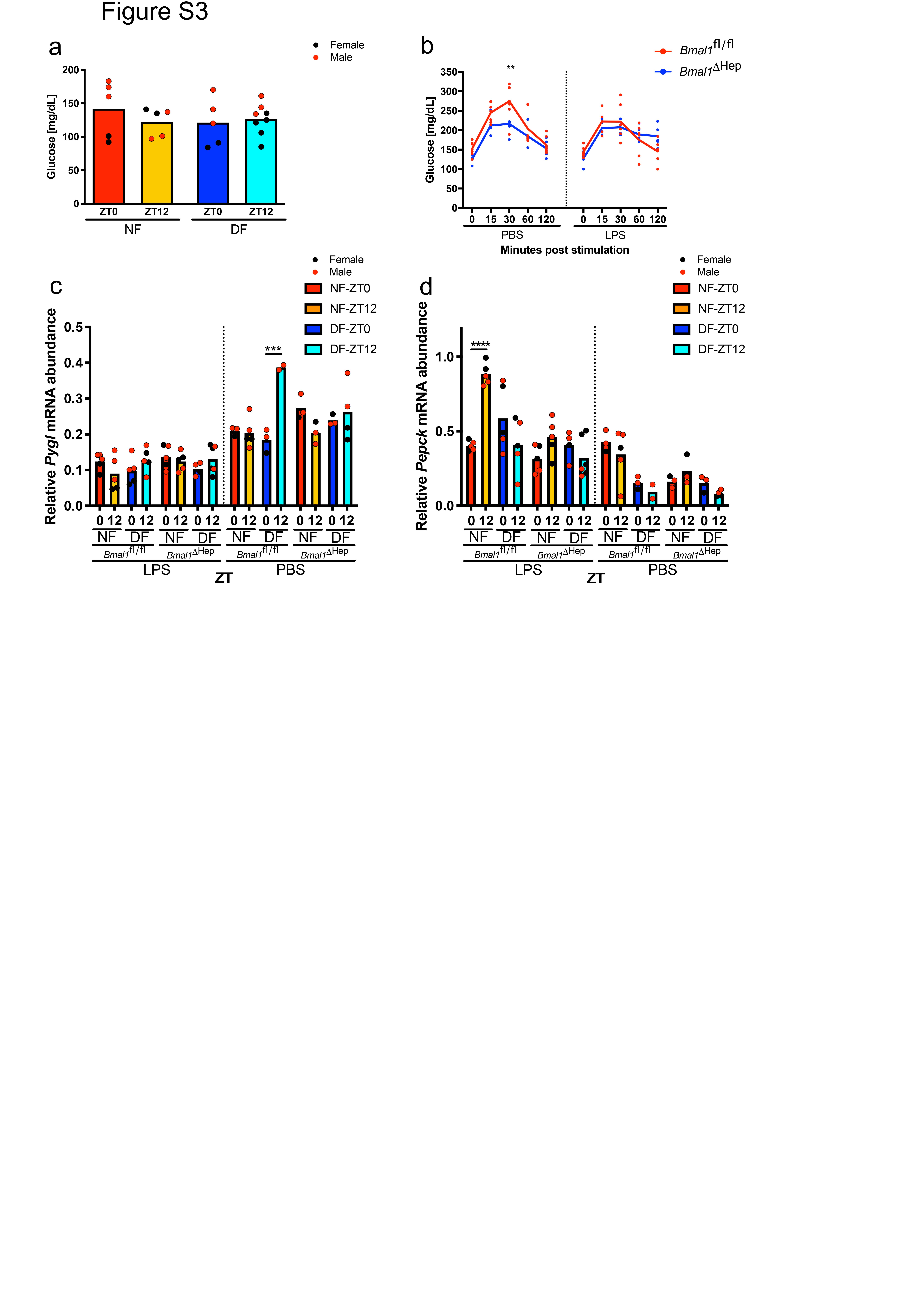


Supplementary Figure 3: **Feeding-regulated** **glucose metabolism is not dependent on the liver clock.** A) Baseline serum glucose on day 5 of TRF at ZT0 or ZT12 as indicated in *Bmal1*^ΔHep^. Female in black, male in red. NF-ZT0 N_female_=2 N_male_=3, NF-ZT12 N_female_=2 N_male_=3, DF-ZT0 N_female_=2 N_male_=3, DF-ZT12 N_female_=5 N_male_=3. Box indicates mean. No statistical significance according to two-way ANOVA. B) PTT on day 5 of day-time feeding at ZT0 in male *Bmal1*^ΔHep^ or *Bmal1*^fl/fl^ control mice co-treated with LPS (20 mg/kg) or PBS at the time of sodium pyruvate (2 g/kg) injection. Two-way ANOVA and Sidak’s multiple comparison. **p=0.0061. LPS: Interaction F_(4,36)_=3.792, p=0.0113, ZT F_(4,36)_=27.35, p<0.0001, Genotype F_(1,9)_=0.005746, p=0.9412. PBS: Interaction F_(4,40)_=1.916, p=0.1265, ZT F_(4,40)_=46.42, p<0.0001, Genotype F_(1,10)_=5.836, p=0.0363. N(*Bmal1*^ΔHep^)=5, N(*Bmal1*^fl/fl^ LPS)=6, N(*Bmal1*^fl/fl^ PBS)=7. Line indicates mean. C, D) Liver *Pygl* (C) or *Pepck* (D) mRNA abundance 6h post LPS (20 mg/kg) or PBS control stimulation at ZT0 or ZT12 as indicated on day 5 of TRF in *Bmal1*^ΔHep^ and *Bmal1*^fl/fl^ control mice. N as shown, each dot represents an individual measurement. Female in black, male in red. Box indicates mean. Two-way ANOVA and Sidak’s multiple comparison (ZT0 vs ZT12). ****p<0.0001, ***p=0.0003. C) LPS: Interaction F_(3,32)_=2.143, p=0.1142, Group F_(1,32)_=0.08411, p=0.7737, Time F_(3,32)_=0.8315, p=0.4864, PBS: Interaction F_(3,18)_=9.237, p=0.0006, Group F_(1,18)_=4.657, p=0.0447, Time F_(3,18)_=3.371, p=0.0414. D) LPS: Interaction F_(3,32)_=12.06, p<0.0001, Group F_(1,32)_=4.616, p=0.0393, Time F_(3,32)_=9.173, p=0.0002, PBS: Interaction F_(3,18)_=0.84, p=0.4896, Group F_(1,18)_=0.847, p=0.3696, Time F_(3,18)_=11.76, p=0.0002.


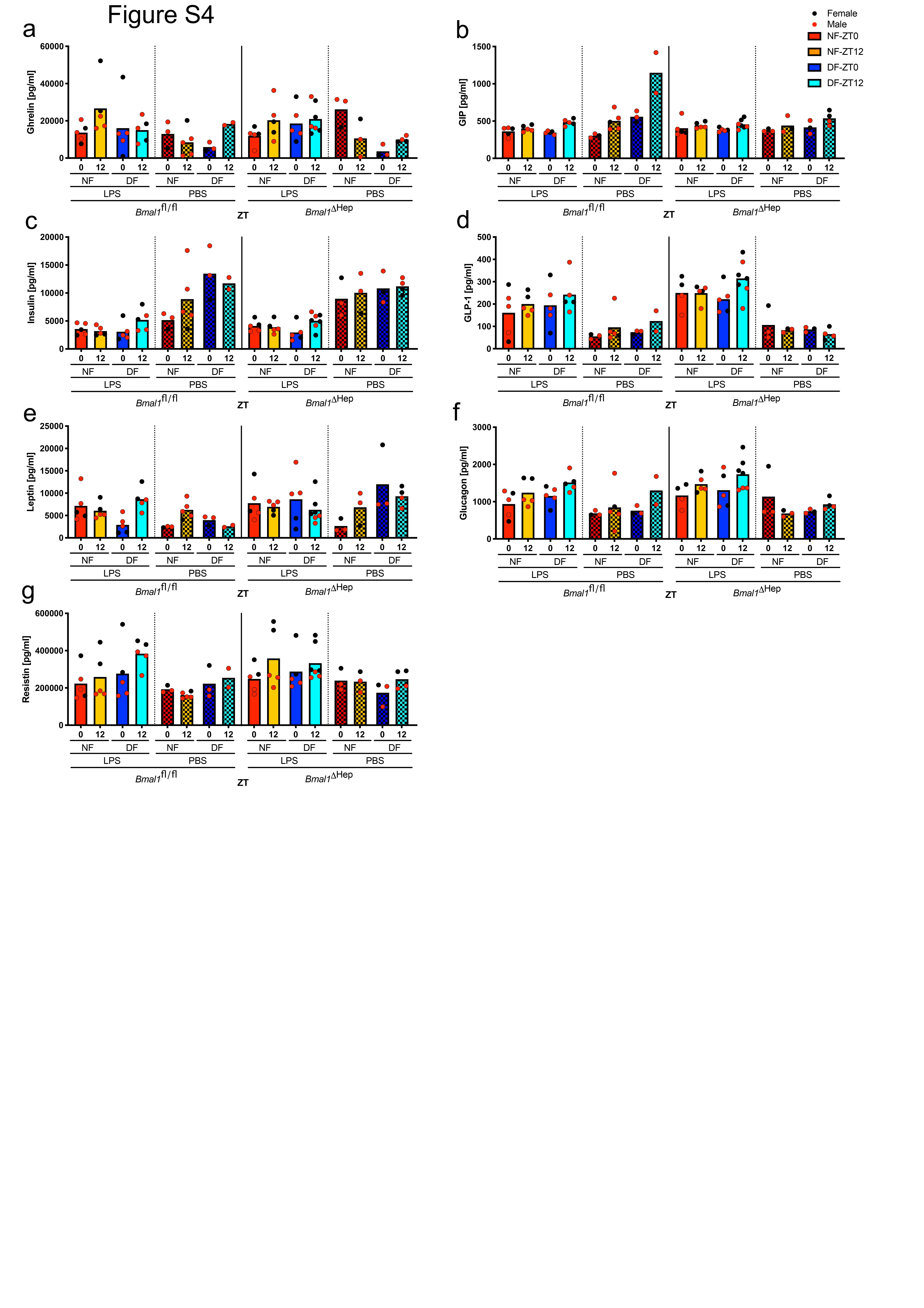


Supplementary Figure 4: **Serum metabolic markers are equally abundant in *Bmal1*^fl/fl^ and *Bmal1*^ΔHep^ mice following LPS stimulation.** A-G) Serum abundance of selected metabolic markers 6 h post LPS stimulation (20mg/kg) or PBS control on day 5 of TRF at ZT0 or ZT12 as indicated in *Bmal1*^fl/fl^ and *Bmal1*^ΔHep^. N as shown, each dot represents an individual measurement. Female in black, male in red. Box indicates mean.


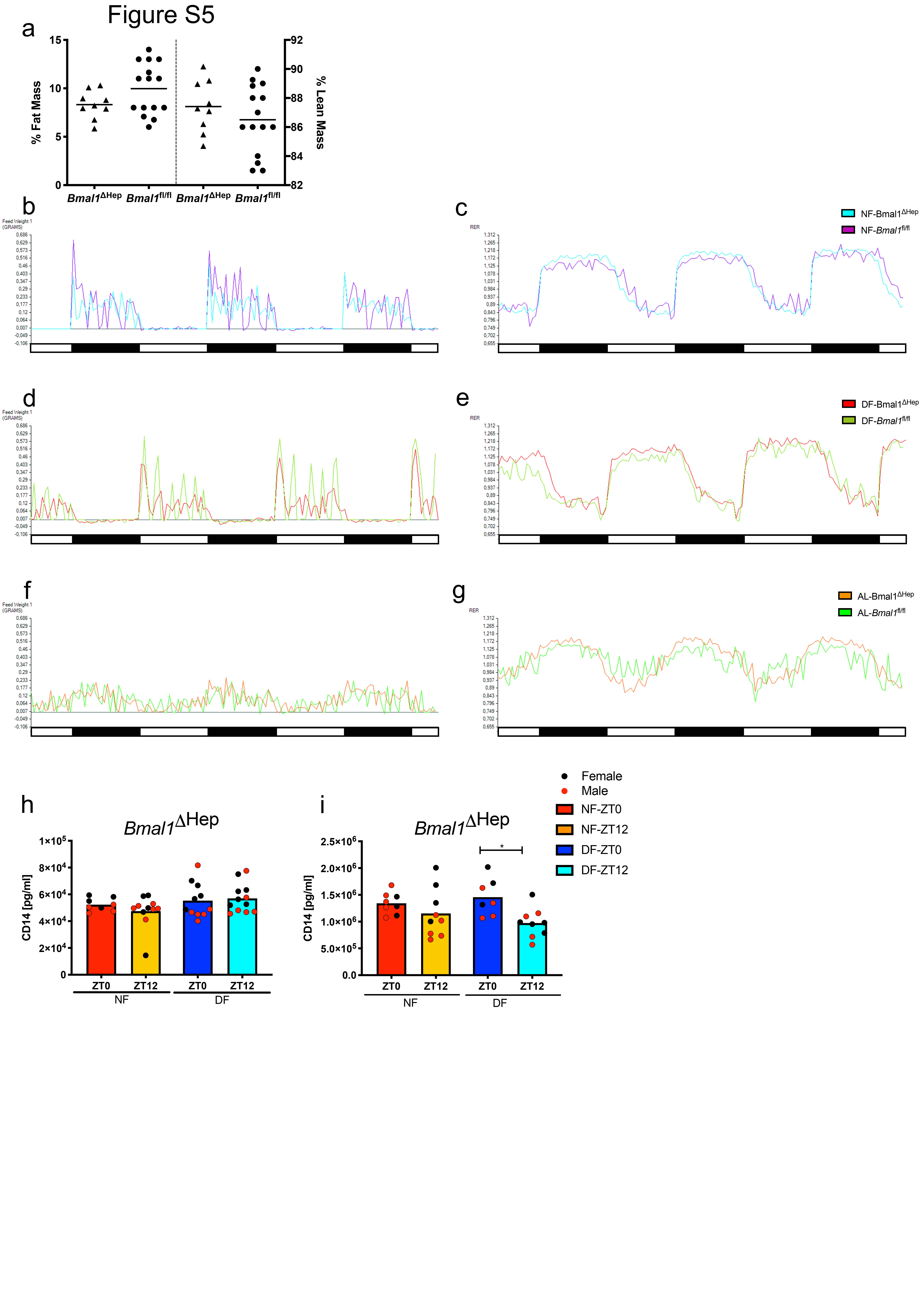


Supplementary Figure 5: **Body composition and feeding phenotype are unaffected by hepatocyte specific *Bmal1* deletion**. A) Percent fat and lean mass of *Bmal1*^ΔHep^ and *Bmal1*^fl/fl^ mice as measured by EchoMRI. No statistical significance between genotypes according to Mann-Whitney. N(*Bmal1*^ΔHep^)=9, N(*Bmal1*^fl/fl^)=15. B-G) Food intake (B, D, F) and RER (C, E, G) of night-time (B, C), day-time (D, E) or *ad libitum* (F, G) fed mice as measured by CLAMS. N(*Bmal1*^ΔHep^)=3, N(*Bmal1*^fl/fl^)=1. H) Baseline serum sCD14 on day 5 of TRF at ZT0, ZT6 or ZT12 as indicated in *Bmal1*^ΔHep^. NF-ZT0 N_female_=4 N_male_=4, NF-ZT12 N_female_=5 N_male_=6, DF-ZT0 N_female_=5 N_male_=6, DF-ZT12 N_female_=6 N_male_=6 . Box indicates mean. Female in black, male in red. No statistical significance using two-way ANOVA. I) Serum sCD14 6h post LPS stimulation (20 mg/kg) on day 5 of TRF at ZT0 or ZT12 as indicated in *Bmal1*^ΔHep^ (p=0.026). NF-ZT0 N_female_=3 N_male_=5, NF-ZT12 N_female_=4 N_male_=5, DF-ZT0 N_female_=3 N_male_=5, DF-ZT12 N_female_=5 N_male_=4. Box indicates mean. Female in black, male in red. Statistical analysis according to two-way ANOVA and Sidak’s multiple comparison as indicated. Interaction F_(1,29)_=1.571, ns, ZT F_(1,29)_=8.324, p=0.0073, feeding F_(1,29)_=0.07527, ns. *p=0.0442.


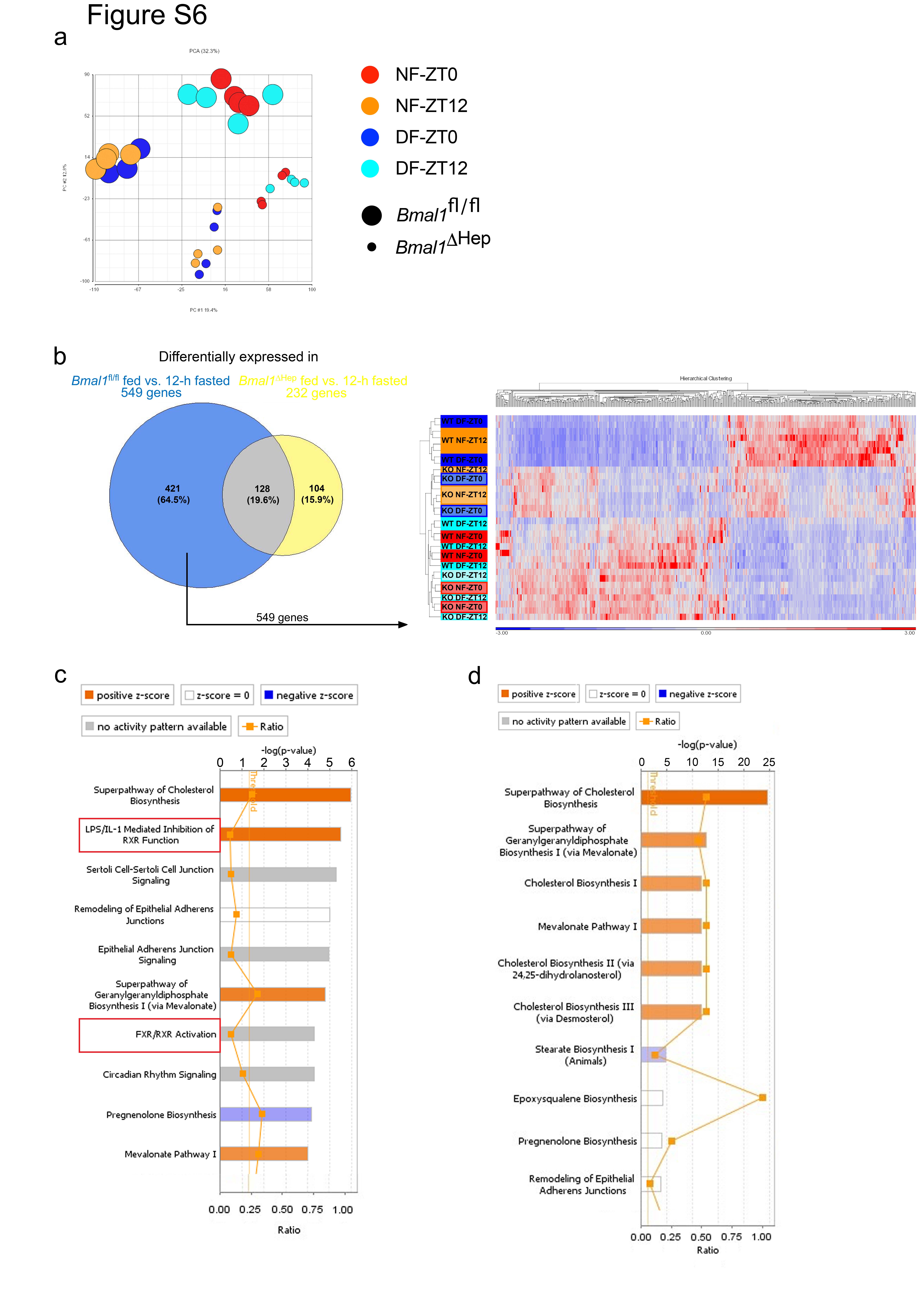


Supplementary Figure 6: **Global hepatic transcriptional changes regulated by the feeding cycle and circadian clock.** A) Principal Component Analysis performed on global gene expression (RPKM) of each animal as measured by liver RNA-Seq on day 5 of TRF at ZT0 or ZT12 as indicated. Group and genotype of each animal shown in different color or size, respectively. N_female_=2 N_male_=2. B) Venn diagram of genes expressed in at least one sample in *Bmal1*^fl/fl^ and *Bmal1*^ΔHep^ liver on day 5 of TRF at ZT0 or ZT12 as indicated, comparing fed groups (NF-ZT0 and DF-ZT12) vs starved groups (NF-ZT12 and DF-ZT0) within each genotype. Genes filtered on those with a FDR <0.2 and at least 2-fold change between fed vs. starved groups within each genotype. Right: Expression profile of 549 genes differentially expressed in the *Bmal1*^fl/fl^ mice between feeding groups subjected to hierarchical clustering and plotted as a heatmap showing relative expression based on RPKM, with expression of the same genes in the other groups of mice shown on the same plot. N_female_=2 N_male_=2. C) Core Analysis using Ingenuity Pathway Analysis of genes that were expressed in at least one animal, a FDR <0.2, and two-fold changed between fed vs. starved groups (n=549) in a one-way ANOVA comparison performed on Log2 transformed RPKM values of *Bmal1*^fl/fl^ liver on day 5 of TRF at ZT0 or ZT12 as indicated, comparing fed (NF-ZT0, DF-ZT12) vs starved (NF-ZT12, DF-ZT0) groups. Top 10 Canonical Pathways are shown. N_female_=2 N_male_=2. D) Core Analysis using Ingenuity Pathway Analysis of genes that were expressed in at least one animal, a FDR <0.2, and two-fold changed between fed vs. starved groups (n=232) in *Bmal1*^ΔHep^ liver on day 5 of TRF at ZT0 or ZT12 as indicated, comparing fed (NF-ZT0, DF12) vs starved (NF-ZT12, DF0) groups in a one-way ANOVA comparison. Top 10 Canonical Pathways are shown. N_female_=2 N_male_=2.

Supplementary Table 1: **CalR based statistical analysis of CLAMS data**


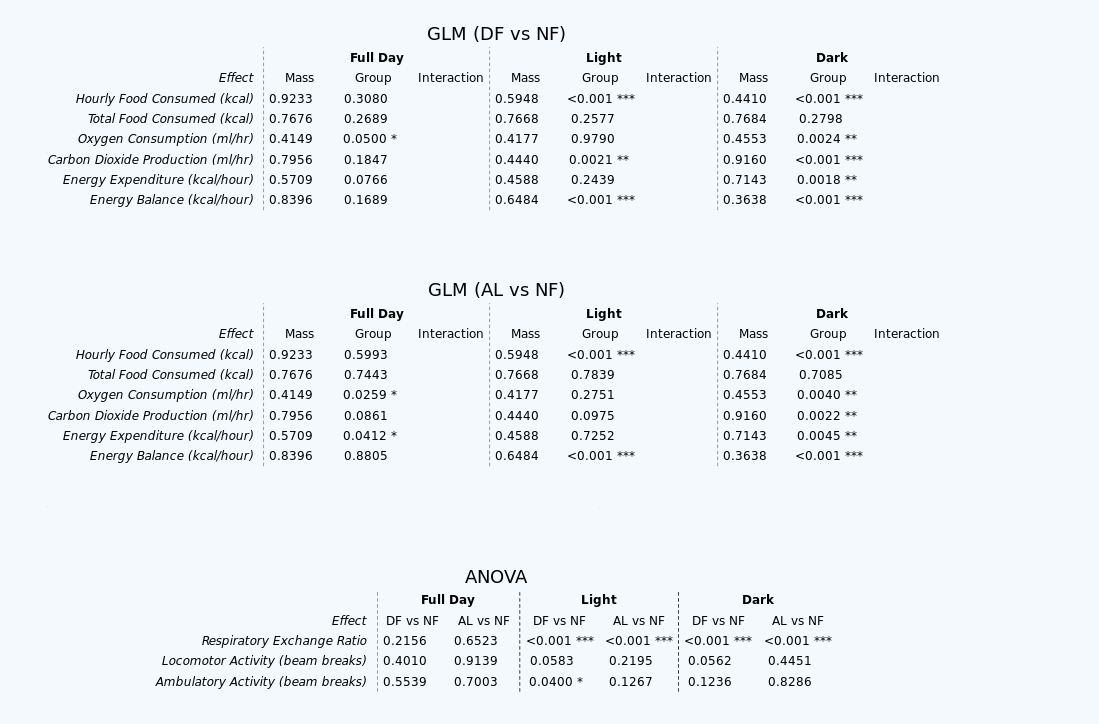


Supplementary Table 2: **Feeding time regulated hepatocyte genes in *Bmal1*^fl/fl^ mice (n=549).**


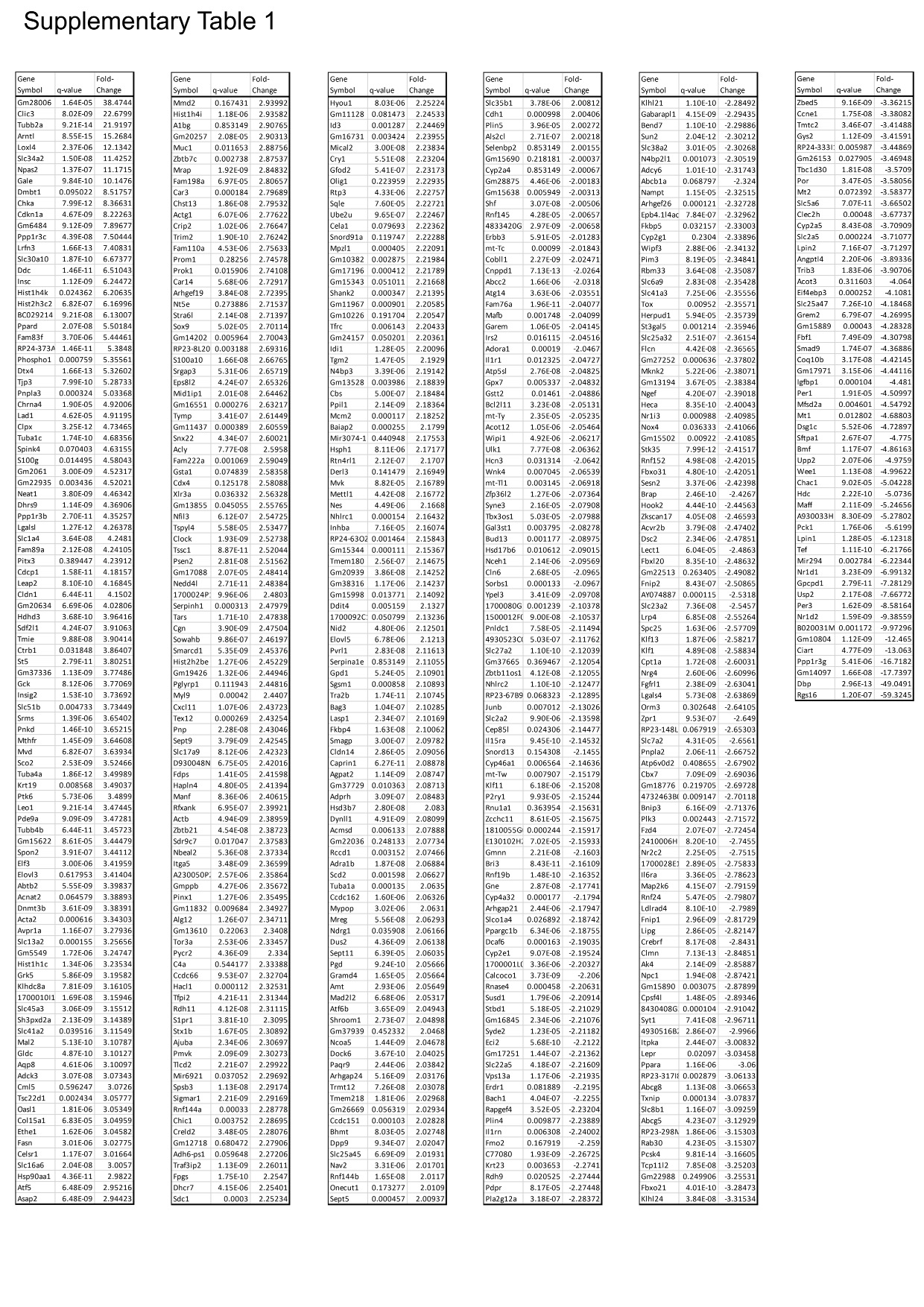


Supplementary Table 3: **Differentially expressed genes in resistant vs. susceptible groups (n=232).**


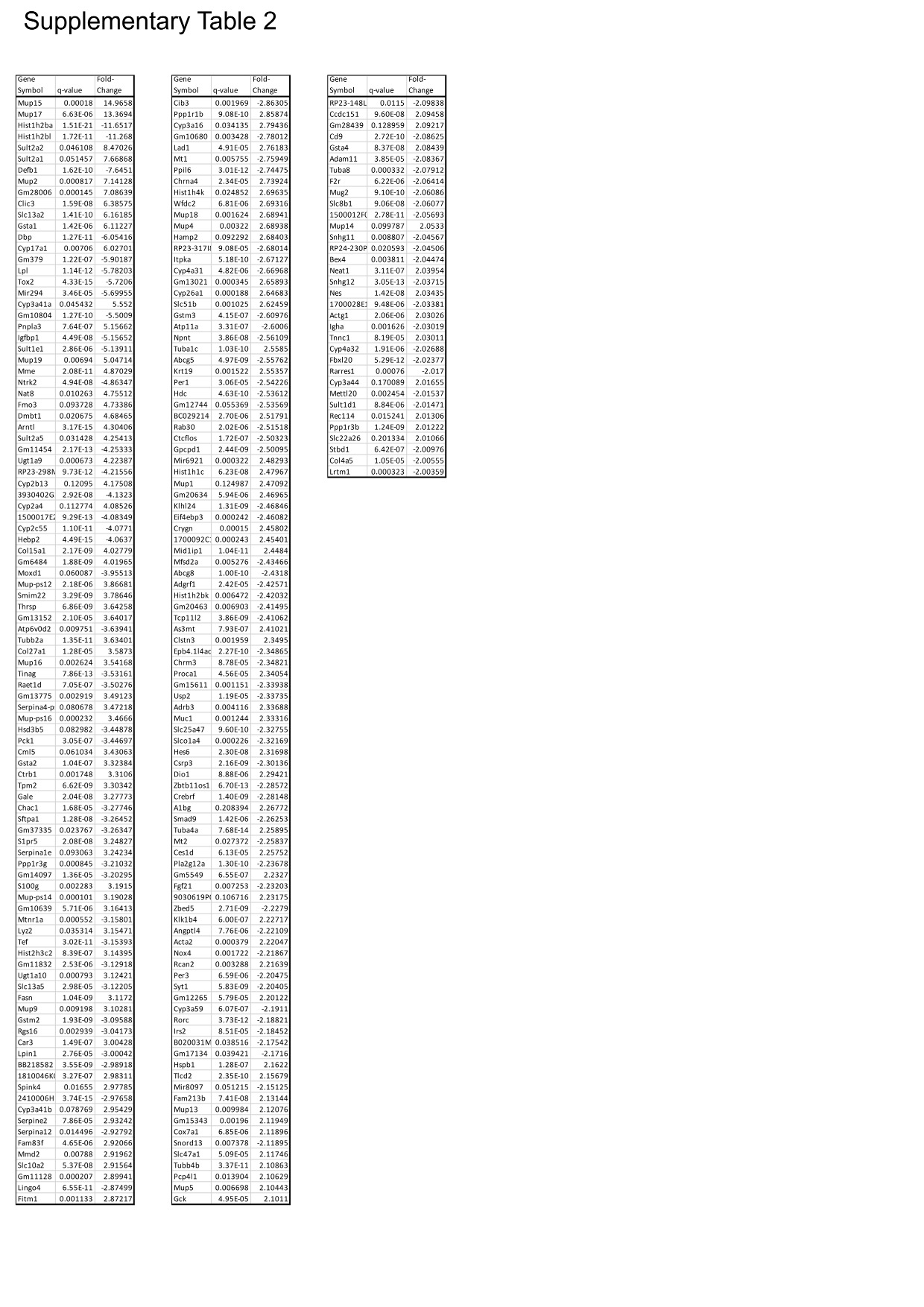


**SUPPLEMENTARY METHODS**

LPS survival scoring

Supplementary Method Table 1: Mortality study scoring sheet.

Mice were continuously scored and killed upon a cumulative score of 8, or a score of 3 in any category.

Supplementary Method Table 2: NanoString CodeSet

Reverse Transcription PCR (RT-PCR) and qPCR

cDNA was prepared from 500 ng RNA using the commercially available kit iScript (BioRad, Cat# 170-8841) according to the manufacturer’s instructions. cDNA was stored at -20 ̊C and diluted 1:5 in ultra-pure water. During the procedure, the RNA and resulting cDNA were kept on ice.

Real time PCR was performed using TaqMan Primer/Probe sets purchased from ThermoFisher (see primer list in Supplementary Method Table 3) and Sso Advanced Universal Probes Supermix (BioRad, Cat# 172-5284) in a CFX384 Touch Real-Time PCR Detection System (BioRad, Cat# 1855485). The cycle number which resulted in a fluorescent signal above threshold (CT) was recorded and data was normalized to the average CT of three housekeeping genes that have previously been validated in a circadian setting (RPS18, Actin, GAPDH) (data not shown).

Supplementary Method Table 2: TaqMan Primer

| gene | ID | cat# | company |
| --- | --- | --- | --- |
| *Pygl* | Mm01289790_m1 | 4331182 | ThermoFisher Scientific |
| *Pepck* (*Pck1*) | Mm01247058_m1 | 4331182 | ThermoFisher Scientific |

References

Kim, I., S. H. Ahn, T. Inagaki, M. Choi, S. Ito, G. L. Guo, S. A. Kliewer, and F. J. Gonzalez. 2007. 'Differential regulation of bile acid homeostasis by the farnesoid X receptor in liver and intestine', *J Lipid Res*, 48: 2664-72.

Mina, A. I., R. A. LeClair, K. B. LeClair, D. E. Cohen, L. Lantier, and A. S. Banks. 2018. 'CalR: A Web-Based Analysis Tool for Indirect Calorimetry Experiments', *Cell Metab*, 28: 656-66 e1.

Wang, L., I. Scott, L. Zhu, K. Wu, K. Han, Y. Chen, M. Gucek, and M. N. Sack. 2017. 'GCN5L1 modulates cross-talk between mitochondria and cell signaling to regulate FoxO1 stability and gluconeogenesis', *Nat Commun*, 8: 523.
